# Evaluation of oncofertility care in childhood cancer patients: the EU-Horizon 2020 twinning project TREL initiative

**DOI:** 10.3389/fped.2023.1212711

**Published:** 2023-07-26

**Authors:** Egle Stukaite-Ruibiene, M. E. Madeleine van der Perk, Goda Elizabeta Vaitkeviciene, Annelies M. E. Bos, Zana Bumbuliene, Marry M. van den Heuvel-Eibrink, Jelena Rascon

**Affiliations:** ^1^ Faculty of Medicine, Vilnius University, Vilnius, Lithuania; ^2^ Princess Máxima Center for Pediatric Oncology, Utrecht, Netherlands; ^3^ Center for Pediatric Oncology and Hematology, Vilnius University Hospital Santaros Klinikos, Vilnius, Lithuania; ^4^ Center of Obstetrics and Gynecology, Vilnius University Hospital Santaros Klinikos, Vilnius, Lithuania

**Keywords:** childhood cancer, fertility counseling, late effects, questionnaire, twinning

## Abstract

**Background:**

The 5-year survival rate of childhood cancer exceeds 80%, however, many survivors develop late effects including infertility. The aim of this study was to evaluate the current status of oncofertility care at Vilnius University Hospital Santaros Klinikos (VULSK) within the framework of the EU-Horizon 2020 TREL project.

**Methods:**

All parents or patients aged 12–17.9 years treated from July 1, 2021 until July 1, 2022 were invited to complete an oncofertility-care-evaluation questionnaire. After completing the questionnaire, patients were triaged to low-risk (LR) or high-risk (HR) of gonadal damage using a risk stratification tool (triage). Data was assessed using descriptive statistics.

**Results:**

Questionnaires were completed by 48 parents and 13 children triaged as 36 (59%) LR and 25 (41%) HR patients. Most HR respondents (21/25, 84%) were not counseled by a fertility specialist. Six boys (4 HR, 2 LR) were counseled, none of the girls was counseled. Three HR boys underwent sperm cryopreservation. Only 17 (27.9%, 9 HR, 8 LR) respondents correctly estimated their risk. All counseled boys (*n* = 6) agreed the risk for fertility impairment had been mentioned as compared to 49.1% (*n* = 27) of uncounseled. All counseled respondents agreed they knew enough about fertility (vs. 42%).

**Conclusions:**

Respondents counseled by a fertility specialist were provided more information on fertility than uncounseled. HR patients were not sufficiently counseled by a fertility specialist. Based on the current experience oncofertility care at VULSK will be improved.

## Introduction

Currently, the 5-year survival rate of patients with childhood cancer exceeds 80%, leading to an increase in the number of childhood cancer survivors (CCS) (1, 2). However, cancer treatment can lead to multiple late toxicities, including gonadal damage and fertility impairment (3–5). Female CCS are at risk for premature ovarian insufficiency, follicular atresia, premature menopause, and infertility, especially after treatment such as alkylating agents and radiotherapy (RT) (6, 7). For male CCS, late effects of chemotherapy or RT to the testes or the hypothalamic–pituitary axis can manifest as hypogonadism and impaired spermatogenesis (8, 9). Fertility is a critical component of life in young CCS, and impaired fertility is associated with reduced long-term quality of life (10, 11). Awareness of potential gonadal damage is crucial for shared decision-making to prevent infertility. Thus, appropriate information and counseling of patients and their caregivers become critical to prevent frustration because of the lack of information.

The International Late Effects of Childhood Cancer Guideline Harmonization Group (IGHG) recommends informing all patients with childhood cancer about their gonadal damage risk and offering fertility counseling to those at risk (12–14). However, implementing these recommendations in clinical practice is challenging. Only a well-established institutional oncofertility care system can ensure timely and adequate counseling of patients with childhood cancer and their caregivers. A five-step oncofertility care plan following IGHG recommendations was developed by the Princess Máxima Center, Netherlands, in 2019 (15). The oncofertility care plan includes the following steps: (1) timely identification of all patients newly diagnosed with childhood cancer, (2) gonadal damage risk stratification using a risk-stratification tool, (3) informing all patients on personal gonadal damage risk (pediatric oncologist or nurse practitioner), (4) counseling the subset at risk (fertility specialist), and (5) offering fertility preservation to children with high gonadal damage risk (HGDR) (15).

The aforementioned oncofertility care plan served as an example to implement IGHG guidelines and improve oncofertility care at the Center for Pediatric Oncology and Hematology of Vilnius University Hospital Santaros Klinikos (VULSK, Lithuania) through a twinning exercise with the Princess Máxima Center within an EU-Horizon 2020-funded project “Twinning in Research and Education to Improve Survival in Childhood Solid Tumors in Lithuania (TREL).” The project aims to foster research on different aspects of pediatric cancer including survivorship (https://siope.eu/TREL-project). A previous cross-sectional study revealed that a majority of adult CCS treated at VULSK had limited knowledge about reproductive health and had not received sufficient information regarding fertility (16). These findings mirror the results of other research groups, suggesting that patients with childhood cancer are not always properly counseled about the effects of cancer treatment on reproductive health (17, 18) or even do not know their fertility status (19).

This study aimed to evaluate the quality of informing and counseling before implementing the oncofertility care plan in an independent cohort of patients with childhood cancer treated at VULSK using an amended version of the tools developed by the Princess Máxima Center—the oncofertility-care-evaluation questionnaires and the gonadal damage risk-stratification tool (triage). We pursued to assess the awareness of patients with childhood cancer or their caregivers of their infertility risk and its compliance with the individual gonadal damage risk assessed by the triage. The results of this study will serve as a baseline for future research.

## Patients and methods

### Oncofertility-care-evaluation questionnaire

First, the oncofertility-care-evaluation questionnaire developed by the Princess Máxima Center was adapted for Lithuanian patients (20). Two questionnaires were used: one for patients counseled by a fertility specialist, and another for those informed by a pediatric oncologist. The questionnaires used a 5-point Likert scale. The design of both questionnaires had been previously published (20). Briefly, the questionnaires were based on multiple validated questionnaires concerning decision regret, reproduction concern, and evaluation of fertility care in an adult setting (21–24). The questions were translated from Dutch to English and afterward from English to Lithuanian. To validate the Lithuanian translation, the reverse translation from Lithuanian to English was performed. No significant discrepancies between the wordings were found. The Lithuanian version was reviewed by two pediatric oncologists, a gynecologist, two patients, and parents, who were all native Lithuanian speakers and fluent in English. Lastly, the Lithuanian version was compared with the Dutch version with help of the English translation by a native Dutch-speaking author. The respondents received the Lithuanian version (Supplementary Tables S1, S2). The English version of the questionnaires is provided in Supplementary Tables S3, S4.

### Gonadal damage risk-stratification tool (triage)

Second, the original gonadal damage risk-stratification tool (triage) used in the Netherlands was amended by the replacement of the treatment protocols that differed between the two institutions (Supplementary Table S5). All protocols and treatment arms used at VULSK were reviewed and included in the amended version, whereas protocols that were only used at the Princess Maxima Center were removed from the amended tool.

In addition, the gonadal damage risk for boys was added to the amended tool because the original version focused on girls only (15). Gonadal damage risk for boys was defined according to the recently published IGHG guidelines (14). The risk of gonadal damage was assessed according to the Cyclophosphamide Equivalent Dose (CED) originally developed by Green et al. (25). The CED scores were classified as low gonadal damage risk (LGDR) (<4,000 mg/m^2^) and HGDR (>6,000 mg/m^2^) for girls (26) and LGDR (<4,000 mg/m^2^) or HGDR (≥4,000 mg/m^2^) for boys (26) (Supplementary Table S5). In the original tool, intermediate gonadal damage risk for girls was defined; however, after the amendment, the gonadal damage risk for girls was classified as low or high according to the newest recommendations (13). In addition, hematopoietic stem cell transplantation, total body irradiation, and RT to the gonads upgrade toward HGDR for boys and girls (13, 14). Boys aged >12 years and Tanner stage >G2P2 should be offered semen cryopreservation (14). All changes from the original gonadal damage risk-stratification tool are summarized in Supplementary Table S6.

### Patients

All parents of patients with childhood cancer (irrespective of the child's age) or patients aged 12–17.9 years treated for pediatric cancer (ICD-10-AM C00-96) between July 1, 2021, and July 1, 2022, were invited to participate in the study. Patients were identified in the institutional database. All patients diagnosed within the evaluation period and those diagnosed previously but still undergoing treatment were invited.

Baseline characteristics including the age at diagnosis, time from the date of diagnosis to enrollment, malignancy type, and treatment (protocol and arm) were retrieved from the medical records. As recorded in the patient files, the date of diagnosis was defined as the date of communicating the cancer diagnosis by a pediatric oncologist to the family.

Participants were divided into two groups: a group counseled by a fertility specialist and a group informed by a pediatric oncologist. Before the start of treatment, every patient received written information on potential risk for fertility impairment (without pointing out a specific individual gonadal damage risk) during the provision of information on chemotherapy and its potential side effects. Thus, all participants not counseled by a fertility specialist were considered informed about the risk for infertility by a pediatric oncologist.

Fertility counseling and fertility preservation were both provided before chemotherapy to all patients at the Santaros Fertility Center, a specialized unit at VULSK. Pubescent boys were counseled by an embryologist based on the results of the semen analysis. During the evaluation period, fertility preservation was only allowed for children aged ≥14 years according to the Lithuanian Law for Assisted Reproduction (26). Therefore, prepubescent girls and boys and their parents were not offered fertility preservation. In cases of fertility preservation, sperm samples were obtained by masturbation, and sperm cryopreservation was performed. If fertility preservation is needed in girls, they are counseled by a gynecologist at VULSK.

### Study outline

A cross-sectional survey within the previously specified timeframe was performed. All participants completed the oncofertility-care-evaluation questionnaire. All patients were already receiving treatment when the questionnaire was handed in, no questionnaires were given between diagnosis and treatment initiation. The questionnaire was completed during the inpatient stay or the scheduled outpatient visit. Questionnaires were completed separately by patients or their parents, and no parent–child combinations were included. As mentioned above, two questionnaires were used: one for patients counseled by a fertility specialist (counseled group), and another for those informed by a pediatric oncologist alone (uncounseled group). The responses were evaluated in July 2022. The responses of the counseled group opposed those of the uncounseled group. The responses to each question were analyzed, and the frequencies of the responses were calculated.

The respondents were also asked to self-estimate their specific risk for infertility. Thereafter, each response was compared with the infertility risk assessment provided by the study team using the above-mentioned risk-stratification tool.

After completing the questionnaires, the respondents were retrospectively triaged to LGDR or HGDR using the amended version of the gonadal damage risk-stratification tool. Data were analyzed in September 2022.

### Statistical analyses

Descriptive statistics were used for all quantitative analyses. The normality of the distribution of continuous variables was evaluated using the Shapiro–Wilk test and expressed as medians with interquartile (IQR) and minimal–maximal ranges. SPSS Statistics version 17 (SPSS Inc., Chicago, IL, USA) was used for all quantitative analyses.

## Results

**Figure 1:**
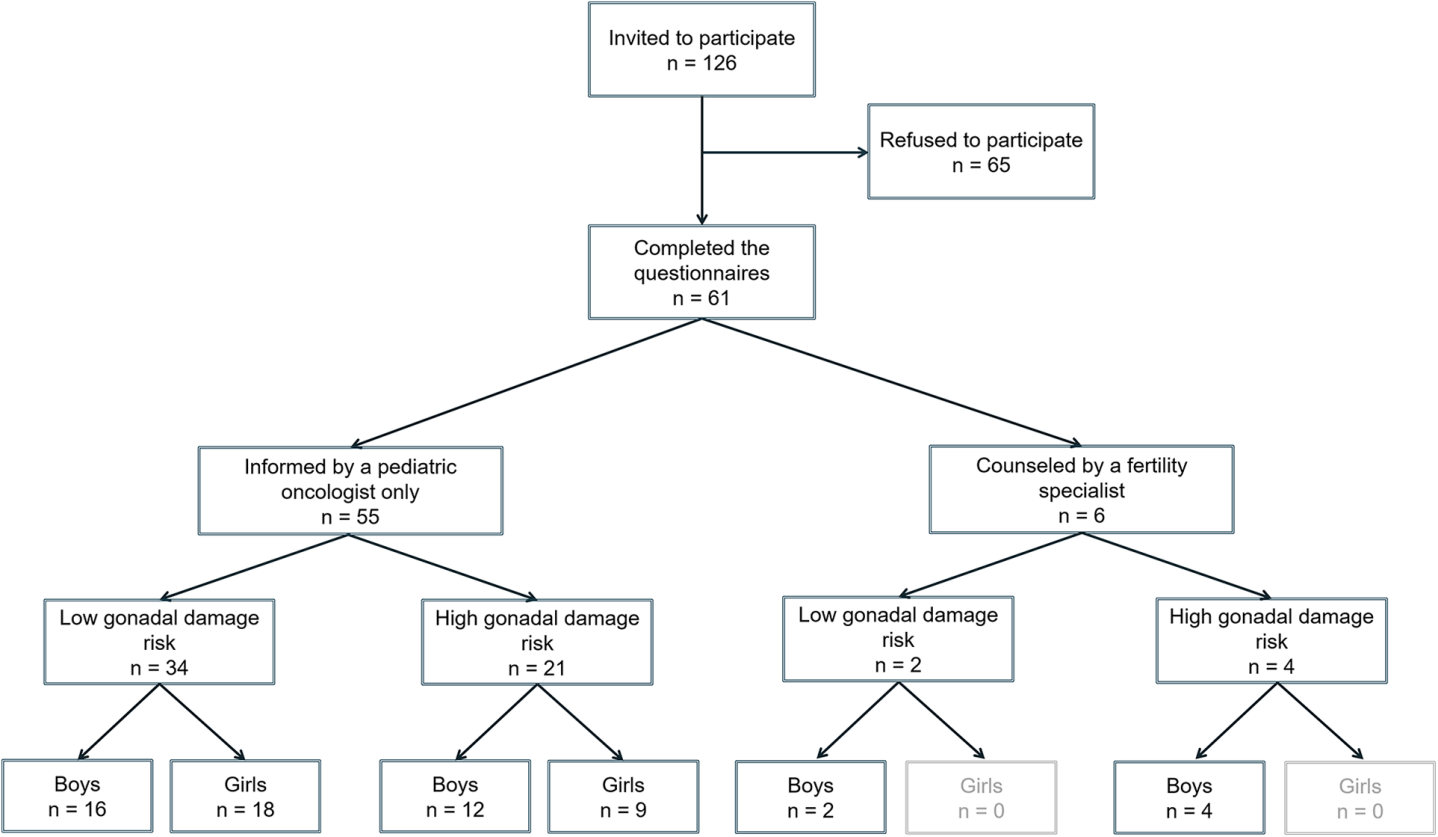
Study flowchart.

In total, 126 patients were invited to participate in the study that evaluated the existing oncofertility (Figure 1). Of the 126 patients, 61 (48.4%) signed the informed consent and were included in the study. All respondents (*n* = 61) completed the oncofertility-care-evaluation questionnaire. More than half of the invited patients (65/126, 51.6%) refused to participate without specifying the reason for refusal. Therefore, their data were not analyzed.

Overall, the questionnaires were completed by 48 parents and 13 children. After completing the questionnaires, the respondents were retrospectively triaged by the study team as 36 LGDR and 25 HGDR. More than half (*n* = 16, 64%) of all HGDR patients were boys and 9 (36%) were girls. Of all 61 respondents, 6 (9.8%) boys were assigned to the counseled group (Figure 1). They were counseled by a fertility specialist (embryologist) before the initiation of cancer treatment, and all completed the questionnaire themselves. After completing the questionnaire, the counseled boys were retrospectively classified as 4 HGDR and 2 LGDR. In the three counseled HGDR boys, normospermia was observed, and sperm cryopreservation was performed. Three boys (2 LGDR, 1 HGDR) did not manage to collect a sperm sample, probably because of their young age.

Of the 21 HGDR respondents in the uncounseled group, 15 (71.4%) were <14 years old; therefore, fertility preservation was not an option as per the legal framework enforced at the time of the study. However, fertility preservation was not offered to six HGDR respondents aged >14 years. Two of them were diagnosed before this study, and three HGDR boys and one HGDR girl aged ≥14 years were diagnosed during the evaluation period.

In the uncounseled group, 55 respondents (48 parents and 7 children) completed the questionnaire (Figure 1). Of these 55 uncounseled respondents, 21 were retrospectively triaged as HGDR and 34 as LGDR. All 55 uncounseled respondents were informed about a potential risk (without pointing out a specific risk) for infertility when consenting to chemotherapy before treatment initiation.

The median age of the respondents at the time of diagnosis was 8 (IQR 4–11) years, which ranged from 3 months to 17 years (Table 1). The median time after diagnosis to enrollment was 16 (IQR 7–23) months in the uncounseled group and 3.5 (IQR 2–6) months in the counseled group. The most common diagnoses were acute lymphoblastic leukemia (ALL) in 21 (34.4%) cases, followed by central nervous system (CNS) tumors in 6 (9.8%), and Hodgkin's lymphoma in 6 (9.8%).

**Table 1:** Characteristics of the study participants according to gonadal damage risk group and diagnosis.

Risk of gonadal damage	Informed by a pediatric oncologist only			Counseled by a fertility specialist			All		
Baseline characteristics	Low (*n* = 34)	High (*n* = 21)	Total (*n* = 55)	Low (*n* = 2)	High (*n* = 4)	Total (*n* = 6)	Low (*n* = 36)	High (*n* = 25)	Total (*n* = 61)
Age at diagnosis (years)	7	6	7	15	17	17	8	7	8
Median (min-max, [IQR])	(1–17, [4–10])	(0.4–16, [3–13])	(0.4–17, [4–11])	(17, [14]	(15–17, [16–17])	(14–17, [15–17])	(1–17, [4–11])	(0.4–17, [4–14])	(0.4–17, [4–14])
Age at enrolment (years)	9	8	8	16	17	17	9	9	9
Median (min-max, [IQR])	(2–18, [6–12])	(0.7–18, [5–15])	(0.7–18, [7–12])	(17 [14])	(16–17, [17])	(16–17) (14–17)	(2–18, [6–12])	(0,7–18, [5–15])	(0.7–18, [5–15])
Time after diagnosis to enrolment (months)	15	18	16	3	5	3.5	14	12	13
Median (min-max, [IQR])	(0.6–37, [8–22])	(0.1–60, [4–28])	(0.1–60, [7–23])	(4, [1])	(0.2–11, [2–8])	(0.2–11, [2–6])	(0.6–37, [7–21])	(0.1–60, [4–22])	(0.1–60, [6–23])
Diagnosis, *n* (%)									
Hematologic malignancies	22 (64.7)	9 (42.9)	31 (56.4)	2 (100)	1 (25)	3 (50)	24 (66.7)	10 (40)	34 (55.7)
Acute lymphoblastic leukemia	18 (52.9)	3 (14.3)	21 (38.2)	-	-	-	18 (50)	3 (12)	21 (34.4)
Acute myeloid leukemia	1 (2.9)	-	1 (1.8)	-	1 (25)	1 (16.7)	1 (2.8)	1 (4)	2 (3.3)
Hodgkin lymphoma	1 (2.9)	4 (19)	5 (9.1)	1 (50)	-	1 (16.7)	2 (5.6)	4 (16)	6 (9.8)
Non-Hodgkin lymphoma	1 (2.9)	2 (9.5)	3 (5.5)	1 (50)	-	1 (16.7)	2 (5.6)	2 (8)	4 (6.6)
Langerhans cell histiocytosis	1 (2.9)	-	1 (1.8)	-	-	-	1 (2.8)	-	1 (1.6)
Solid tumors	9 (26.5)	9 (42.9)	18 (32.7)	-	3 (75)	3 (50)	9 (25)	12 (48)	21 (34.4)
CNS tumors	3 (8.8)	3 (14.3)	6 (10.9)	-	-	-	3 (8.3)	3 (12)	6 (9.8)
Neuroblastoma	2 (5.9)	2 (9.5)	4 (7.3)	-	-	-	2 (5.6)	2 (8)	4 (6.6)
Renal tumors	4 (11.8)	1 (4.8)	5 (9.1)	-	-	-	4 (11.1)	1 (4)	5 (8.2)
Osteosarcoma	2 (5.9)	-	2 (3.6)	-	-	-	2 (5.6)	-	2 (3.3)
Ewing sarcoma	-	1 (4.8)	1 (1.8)	-	1 (25)	1 (16.7)	-	2 (8)	2 (3.3)
Soft tissue sarcoma	1 (2.9)	3 (14.3)	4 (7.3)	-	1 (25)	1 (16.7)	1 (2.8)	4 (16)	5 (8.2)
Germ cell tumor	-	1 (4.8)	1 (1.8)	-	1 (25)	1 (16.7)	-	2 (8)	2 (3.3)
Retinoblastoma	-	1 (4.8)	1 (1.8)	-	-	-	-	1 (4)	1 (1.6)
Total	34	21	55	2	4	6	36	25	61

**Figure 2:**
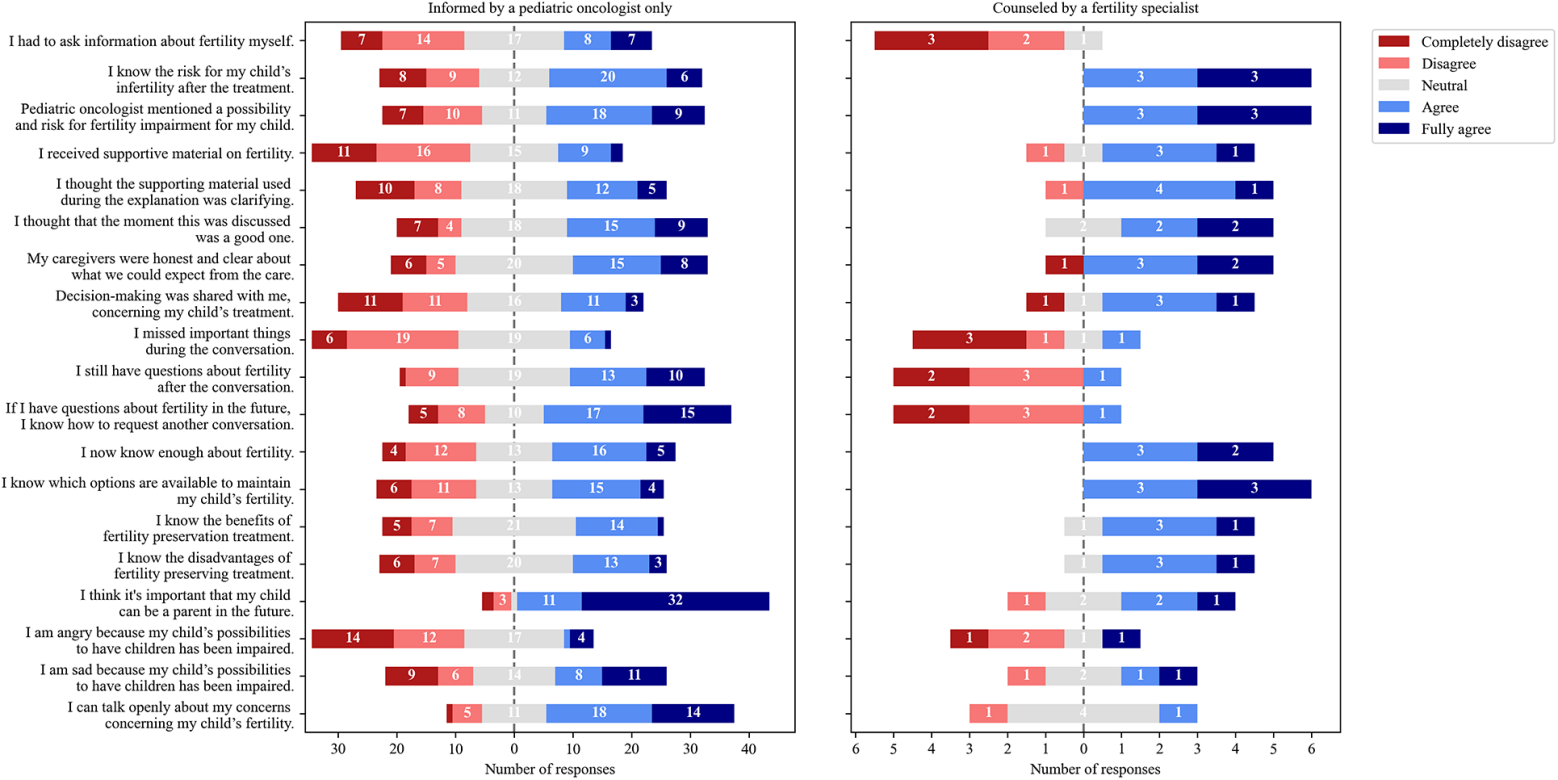
Distribution of answers of respondents informed by a pediatric oncologist only (*n* = 55) and those additionally counseled by a fertility specialist (*n* = 6).

The counseled group (*n* = 6, 4 HGDR, 2 LGDR) indicated more often that they were provided more information on fertility than the uncounseled group (*n* = 55, 21 HGDR, 34 LGDR) (Figure 2). All six counseled boys stated that the risk for fertility impairment was mentioned compared with 49.1% of uncounseled respondents (*n* = 27). The counseled group reported that they now know enough about fertility (vs. *n* = 21, 42%) and knew options for fertility preservation (vs. *n* = 19, 38.8%). Only 10/55 (19.2%) of uncounseled respondents received supportive material on fertility (vs. *n* = 4, 66.7% counseled). All responses of the uncounseled and counseled groups are provided in Supplementary Figures S1, S2.

Quite a few (31/61, 50.8%) respondents commented to the open question, “What time do you think is the best time to have a conversation about fertility?” Most respondents (27/31, 87.1%) answered that fertility should be discussed before initiating cancer treatment. Four uncounseled respondents (2 HGDR, 2 LGDR) thought the best time would be after the treatment. Some (17/55, 30.9%) uncounseled respondents stated they never had a conversation about fertility.

The majority (44/61, 72.2%) of respondents could not correctly self-estimate their specific gonadal damage risk in comparison with the risk allocated by the study team (Table 2). In total, only 17/61 (27.9%) respondents correctly estimated their gonadal damage risk (9 HGDR, 8 LGDR). Of the uncounseled respondents, 22/55 (40%) reported that they do not know their risk, whereas 14/55 (25.5%) correctly estimated their risk. Interestingly, only three counseled boys (2 HGDR, 1 LGDR) correctly estimated their risk.

**Table 2:** Comparison of the gonadal damage risk estimated by the gonadal damage risk-stratification tool (triage) and gonadal damage risk according to the respondents (self-evaluation).

Risk of gonadal damage according to the triage^a^	Informed by a pediatric oncologist only (*n* = 55)		Counseled by a fertility specialist (*n* = 6)	
Risk of gonadal damage according to the respondents^b^	Low	High	Low	High
Low	7	2	1	1
High	2	7	-	2
I don't know	22	12	1	1
Did not answered	3	-	-	-

^a^ Estimated by an oncofertility care team.

^b^ Estimated by 48 parents and 13 children who completed the questionnaires.

## Discussion

The increasing number of CCS raises the need for focusing the research on the late effects of cancer treatment and the well-being of CCS. Gonadal damage risk and fertility counseling have beneficial effects on the quality of life of CCS regardless of the decision on fertility preservation (27, 28). Thus, gonadal toxicity must be discussed before the initiation of any therapy (29–32). In our previous cross-sectional study, a majority of adult CCS who were in a long-term remission (age 18+ years as of December 2016) treated at VULSK had limited knowledge about reproductive health and did not receive sufficient information regarding fertility (16). At the time of this previous study patients did not get any information on fertility damage mostly because survival rate was quite low and long-term treatment effects were not a primary concern. Besides, fertility preservation at that time was not possible. From the previous study, we learned that potential azoospermia after high alkylating agents dosages should imply semen preservation before treatment (16). The results prompted the initiation of counseling on fertility damage (6 boys were counseled in this study) and building up the fertility preservation services (performed for 4 boys in this study) (Figure 1). As it was started to provide fertility care, an evaluation of what patients consider appropriate care, including the effect of receiving information and counseling toward fertility preservation, is essential to improve oncofertility care and the quality of life of CCS.

To achieve an essential breakthrough in oncofertility and survivorship care at VULSK, research collaboration with Princess Máxima Center was initiated as part of the EU-funded HORIZON 2020 twinning project TREL. As previously mentioned, a five-step oncofertility care plan launched at the Princess Máxima Center in 2019 served as a model (15). Until recently, fertility information and counseling lacked a systematic approach. Patients were referred to a fertility specialist sporadically when high infertility risk was suspected as there were no tools developed for the evaluation of infertility risk. Moreover, there were no strict selection criteria for referral to a specialist counseling. Therefore, a slightly amended oncofertility care plan was implemented at VULSK in July 2022. Fertility preservation is quite a new topic in Lithuania as the Law for Assisted Reproduction was only adopted in 2017 (26).

During the evaluation of oncofertility care, the most common tumors of the study participants were ALL, CNS tumors, and lymphomas (Table 1), reflecting the international incidence of childhood cancer (33). Boys were more frequently stratified as having HGDR than girls (Figure 1). This is an expected finding because a lower dose of alkylating agents (4,000 mg/m^2^) leads to HGDR for boys compared with girls (26, 26).

All respondents were triaged retrospectively, i.e., after completing the questionnaire. However, triaging and informing patients about their specific gonadal damage risk are recommended before the start of cancer treatment. However, this is not feasible for some cancer types, e.g., most patients with ALL are assigned to a risk-appropriate treatment arm after the induction therapy (15). The gonadotoxic treatment has already been initiated before the definitive treatment intensity is known. Thus, the application of the timely triage definition to patients with ALL, acute myeloid leukemia (AML), and non-Hodgkin's lymphoma (NHL) in clinical practice is challenging. These patients should be informed about a potential gonadal damage risk before the treatment. However, the definitive triage should be postponed until complete remission or treatment arm allocation because the final intensity of the front-line treatment in hematologic malignancies is assigned after the induction therapy. In patients with renal tumors, delaying the triage after surgery when definitive treatment stratification, including optional RT, will be determined would be beneficial.

In this study, only six (4 HGDR, 2 LGDR) boys were counseled by a fertility specialist (Figure 1). As mentioned above, the counseling and fertility preservation rate was low because, at the time of the evaluation, fertility preservation was only allowed for children aged ≥14 years according to the Lithuanian legislation (26). Fortunately, the legal basis was changed on July 1, 2022, and currently, fertility-preservation options are available to all children irrespective of age. As recommended, we aimed to offer sperm cryopreservation to all postpubertal and pubertal boys aged ≥14 years diagnosed with childhood cancer (26). The procedure is quite simple and non-invasive and does not postpone the cancer treatment (34). In addition, previous reports have shown that sperm cryopreservation in male adolescents with childhood cancer is underused (35). Contrastingly, harvesting testicular tissue for cryopreservation is still considered an experimental technique (26). Moreover, there is a high risk of reintroducing cancer cells during autotransplantation in patients with Hodgkin's lymphoma, NHL, ALL, and AML (36, 37). At present, ovarian tissue cryopreservation is considered standard care for prepubertal and peripubertal girls (15, 38). However, future autotransplantation of ovarian tissue from children is still being investigated and considered experimental (39). Oocyte cryopreservation is another established method for fertility preservation in postpubertal girls and young female adults; nevertheless, it could delay the initiation of cancer treatment (26). This was the reason why an HGDR girl aged ≥14 years diagnosed during the evaluation period was not counseled by a gynecologist. However, after the implementation of the oncofertility care plan, all girls at VULSK will be offered the possibility of fertility preservation as recommended (26).

The self-evaluation exercise revealed that the majority (36/61, 59%) of the respondents did not know their specific gonadal damage risk (Table 2). All counseled respondents (*n* = 6) shared that they know enough about fertility (Figure 2); however, only 3/6 (50%) correctly estimated their risk (Table 2). Results suggest that the gonadal damage risk should be communicated to the patients more clearly and even repeatedly. Of the uncounseled respondents, 27/55 (49.1%) stated that a possibility and risk for fertility impairment was mentioned compared all counseled respondents (Figure 2). Approximately a third of uncounseled respondents stated they did not have a conversation about fertility at all. As mentioned, during this study, all patients received written information on potential side effects of treatment, and gonadal damage was mentioned along with other potential toxicities. The statement that fertility was not mentioned should be interpreted carefully because studies have shown that patients and parents do not remember all the information provided during stressful situations and only 20% of information is possibly retained (40, 41). In addition, the median time after diagnosis to enrollment was longer for the uncounseled respondents than for the counseled ones (16 vs. 3.5 months) (Table 1). Considering that time had elapsed, uncounseled respondents had probably forgotten some information. In addition, results revealed a lack of provision of supportive materials, and only 10/55 (19.2%) uncounseled respondents reported that they received supportive material (Figure 2). Thus, informative leaflets were produced, which will be handed out to every patient.

According to the IGHG guidelines, a pediatric oncologist, endocrinologist, fertility specialist, or a specialized nurse may conduct fertility counseling (26). Our results confirm again that counseling by a fertility specialist differs from informing by a pediatric oncologist, and in high-risk patients, after being informed, counseling by an expert must be considered (Figure 2). However, in this study, counseled respondents included boys only.

The low total number of respondents and counseled respondents could be a major study limitation. The number of study participants reflects the annual patient volume at VULSK, i.e., approximately 50–60 new patients with childhood cancer are diagnosed and treated annually (there are two pediatric oncology centers in Lithuania for the 2.8 million population) (20). Considering the low number of patients, all patients who were treated during the evaluation period were invited to participate, including both new and previously diagnosed patients. On account of the low number of respondents, only a descriptive data analysis was conducted because the results of statistical tests could be misleading. Responses of parents versus patients and boys versus girls were not compared. Only half of the invited patients (61/126, 48.4%) agreed to participate in the study. Therefore, response bias is probably, e.g., patients who were provided enough information regarding reproductive health refused to participate. However, to our knowledge, this is the first study that analyzed how patients with childhood cancer and their parents experience fertility care, being a strength of this study.

The results of this study revealed that fertility care for patients with childhood cancer can be quite difficult in clinical practice. Results revealed a need for daily identification, coordination, and documentation of newly diagnosed, timely triaged, and informed patients. Therefore, the oncofertility care plan was implemented at VULSK using an adapted risk-based oncofertility care system, and its systematic evaluation was implemented at the Princess Máxima Center. Therefore, the experience of fertility care from the points of view of the patients and parents could be compared between the two centers.

This study contributed to the awareness of pediatric oncologists of the gonadal damage risk and facilitated referral to a fertility specialist. The study provides insight to further oncofertility care practice, and the results will contribute to the enhancement of fertility care and possibly aid in improving the quality of life of CCS. The oncofertility care at VULSK will be reevaluated after the implementation of the oncofertility care plan. The twinning activities between the two institutions contributed to the reduction of disparities in oncofertility care and research.

## Conclusions

The existing system of informing patients on gonadal damage risk, along with all other potential side effects of chemotherapy, must be improved. Counseling of children at HGDR by a fertility specialist is important and appears more efficient than informing by a pediatric oncologist, only providing adequate information. The absence of systematic gonadal damage risk stratification before treatment initiation and former restrictions in the Lithuanian legal framework jeopardized sufficient patient empowerment regarding their fertility-preservation options. These baseline results will be used to compare oncofertility care before and after the implementation of the oncofertility care plan. The oncofertility care plan established by the Princess Máxima Center was adopted and is in the process of implementation at VULSK as a part of the twinning project TREL, which will contribute to the reduction of disparities in oncofertility care and research.

## Data Availability

The raw data supporting the conclusions of this article will be made available by the authors, without undue reservation.
