# Supplementary material for: Evaluation of oncofertility care in childhood cancer patients: the EU-Horizon 2020 twinning project TREL initiative
Source: Front Pediatr. 2023 Jul 26;11:1212711. doi: 10.3389/fped.2023.1212711 (PMC10411952; doi:10.3389/fped.2023.1212711)
Supplement: Supplementary file 1 [file Datasheet1.pdf]

Table S1: Lithuanian questionnaire for patients counseled by a fertility specialist.

TREL

Twinning in Research and Education to improve survival in Childhood Solid Tumours in Lithuania

Versija 3.0 2021-06-25

Vilniaus universiteto ligoninėje Santaros klinikose vyksta Horizon 2020 „Mokslinių tyrimų ir švietimo bendradarbiavimo projektas, siekiant pagerinti vaikų, sergančių piktybiniais navikais, išgyvenamumą Lietuvoje (TREL) Nr.952438". Projekto tikslas – pagerinti vaikų, sergančių onkologinėmis ligomis išgyvenamumą ir gyvenimo kokybę pasveikus.

Pasveikus nuo onkologinės ligos gali nukentėti vaisingumas – t .y. galimybė susilaukti vaikų. Vienas iš projekto tikslų – įvertinti, ar pacientų tėvai/globėjai ir patys pacientai tinkamai informuojami apie nevaisingumo riziką.

Prieš kurį laiką Jūsų vaikui buvo diagnozuota onkologinė liga. Prašytume pasidalinti savo patirtimi užpildant šį klausimyną, siekiant pagerinti konsultavimo dėl vaisingumo kokybę.

Aš esu: (pažymėkite ☒)

Vaiko mama ☐ Vaiko tėtis ☐

Auginu:

Mergaitę ☐ Berniuką ☐

Jūsų vaiko nevaisingumo rizika po onkologinės ligos gydymo:

Žema ☐ Vidutinė ☐ Aukšta ☐ Nežinau ☐

Žemiau esantys teiginiai apibūdina konsultaciją dėl Jūsų vaiko vaisingumo. Pasirinkite vieną variantą prie kiekvieno teiginio. Nurodykite, kaip stipriai sutinkate su pateiktu teiginiu, apibraukdami skaičių prie atsakymo (1- visiškai nesutinku, 5 – visiškai sutinku).

Bendri klausimai:

		Visiškai nesutinku	Nesutinku	Nei sutinku, nei nesutinku	Sutinku	Visiškai sutinku
1	Mūsų gydytoja paminėjo vaisingumą pirmoje konsultacijoje, kai buvo aptarta mano vaiko diagnozė.	1	2	3	4	5
2	Kai sužinojau apie diagnozę ir gydymą, susirūpinau dėl savo vaiko vaisingumo.	1	2	3	4	5
3	Turėjau pats(-i) paprašyti suteikti informaciją apie vaisingumą.	1	2	3	4	5

Klausimai, susiję su vaikų onkologo konsultacija:

		Visiškai nesutinku	Nesutinku	Nei sutinku, nei nesutinku	Sutinku	Visiškai sutinku
4	Jaučiau, kad tuo metu buvo svarbu gauti informaciją apie vaisingumą.	1	2	3	4	5
5	Manau, kad pokalbis apie vaisingumą įvyko tinkamu metu.	1	2	3	4	5
6	Man buvo pateikta pagalbinė medžiaga apie vaisingumą (lankstinukai, knygos, nuorodos internete ar kt.)	1	2	3	4	5
7	Pateikta pagalbinė medžiaga buvo suprantama ir aiški.	1	2	3	4	5
8	Informacija apie nevaisingumą man buvo suprantama.	1	2	3	4	5
9	Man trūko svarbių dalykų per pokalbį dėl vaisingumo.	1	2	3	4	5

Klausimai, susiję su ginekologo/urologo konsultacija:

		Visiškai nesutinku	Nesutinku	Nei sutinku, nei nesutinku	Sutinku	Visiškai sutinku
10	Manau, kad konsultacija įvyko tinkamu metu.	1	2	3	4	5
11	Man buvo pateikta pagalbinė medžiaga apie vaisingumą (lankstinukai, knygos, nuorodos internete ar kt.)	1	2	3	4	5
12	Pateikta pagalbinė medžiaga buvo suprantama ir aiški.	1	2	3	4	5
13	Aš žinau, kokia yra nevaisingumo rizika mano vaikui pasibaigus gydymui.	1	2	3	4	5
14	Man buvo papasakota, kokie yra galimi būdai vaisingumo išsaugojimui.	1	2	3	4	5
15	Buvo aptarti vaisingumo išsaugojimo gydymo privalumai.	1	2	3	4	5
16	Buvo aptarti vaisingumo išsaugojimo gydymo trūkumai.	1	2	3	4	5
17	Aš galėjau spręsti dėl savo vaiko vaisingumo ateityje.	1	2	3	4	5

18	Informacija apie nevaisingumą man buvo suprantama.	1	2	3	4	5
19	Man buvo išaiškintos mano vaiko vaisingumo išsaugojimo gydymo galimybės.	1	2	3	4	5
20	Išaiškinimas apie vaisingumo išsaugojimo gydymo galimybes buvo suprantamas.	1	2	3	4	5
21	Gydytojai buvo atviri apie tai, ko galima tikėtis iš vaisingumo priežiūros.	1	2	3	4	5
22	Man trūko svarbių dalykų per konsultaciją dėl vaisingumo.	1	2	3	4	5
23	Po konsultacijos vis dar turiu klausimų apie vaisingumą.	1	2	3	4	5
24	Jeigu turėsiu klausimų apie vaisingumą ateityje, žinau, kur kreiptis.	1	2	3	4	5
25	Galėjau priimti sprendimą dėl savo vaiko vaisingumo išsaugojimo gydymo.	1	2	3	4	5
26	Galėjau rinktis be spaudimo ar kitų daromos įtakos.	1	2	3	4	5

Klausimai, susiję su abiems pokalbiais dėl vaisingumo:

		Visiškai nesutinku	Nesutinku	Nei sutinku, nei nesutinku	Sutinku	Visiškai sutinku
27	Aš esu gerai informuotas(-a) apie vaisingumą.	1	2	3	4	5
28	Aš dabar žinau pakankamai apie vaisingumą.	1	2	3	4	5
29	Aš žinau, kokia mano vaiko nevaisingumo rizika dėl gydymo.	1	2	3	4	5
30	Aš žinau, kokios yra mano vaiko vaisingumo išsaugojimo galimybės.	1	2	3	4	5
31	Aš žinau apie vaisingumo išsaugojimo gydymo privalumus.	1	2	3	4	5
32	Aš žinau apie vaisingumo išsaugojimo gydymo trūkumus.	1	2	3	4	5
33	Aš pykstu, nes sumažėjo mano vaiko galimybės turėti vaikų.	1	2	3	4	5
34	Man svarbu, kad mano vaikas ateityje galėtų būti mama/tėčiu.	1	2	3	4	5

35	Aš galiu atvirai kalbėti apie savo susirūpinimą dėl savo vaiko vaisingumo.	1	2	3	4	5
36	Man liūdna, nes sumažėjo mano vaiko galimybės turėti vaikų.	1	2	3	4	5
37	Aš priėmiau teisingą sprendimą dėl vaisingumo išsaugojimo.	1	2	3	4	5
38	Aš gailiuosi priimo sprendimo.	1	2	3	4	5
39	Dabar aš priimčiau kitokį sprendimą.	1	2	3	4	5

Ar yra dalykų, kurių jums trūko per pirmą pokalbį ar konsultaciją dėl vaisingumo?

Kaip manote, kada yra tinkamiausias laikas pokalbiui apie vaisingumą?

Kaip manote, kas praėjo gerai per Jūsų vaiko vaisingumo priežiūrą?

Ar turite kitų pastebėjimų ar patarimų dėl vaisingumo priežiūros?

Ačiū už skirtą laiką!

Table S2: Lithuanian questionnaire for patients informed by a pediatric oncologist only.

TREL

Twinning in Research and Education to improve survival in Childhood Solid Tumours in Lithuania

Versija 3.0 2021-06-25

Vilniaus universiteto ligoninėje Santaros klinikose vyksta Horizon 2020 „Mokslinių tyrimų ir švietimo bendradarbiavimo projektas, siekiant pagerinti vaikų, sergančių piktybiniais navikais, išgyvenamumą Lietuvoje (TREL) Nr.952438". Projekto tikslas – pagerinti vaikų, sergančių onkologinėmis ligomis išgyvenamumą ir gyvenimo kokybę pasveikus.

Pasveikus nuo onkologinės ligos gali nukentėti vaisingumas – t .y. galimybė susilaukti vaikų. Vienas iš projekto tikslų – įvertinti, ar pacientų tėvai/globėjai ir patys pacientai tinkamai informuojami apie nevaisingumo riziką.

Prieš kurį laiką Jūsų vaikui buvo diagnozuota onkologinė liga. Prašytume pasidalinti savo patirtimi užpildant šį klausimyną, siekiant pagerinti konsultavimo dėl vaisingumo kokybę.

Aš esu: (pažymėkite ☒)

Vaiko mama ☐ Vaiko tėtis ☐

Auginu:

Mergaitę ☐ Berniuką ☐

Jūsų vaiko nevaisingumo rizika po onkologinės ligos gydymo:

Žema ☐ Vidutinė ☐ Aukšta ☐ Nežinau ☐

Žemiau esantys teiginiai apibūdina konsultaciją dėl Jūsų vaiko vaisingumo. Pasirinkite vieną variantą prie kiekvieno teiginio. Nurodykite, kaip stipriai sutinkate su pateiktu teiginiu, apibraukdami skaičių prie atsakymo (1- visiškai nesutinku, 5 – visiškai sutinku).

Bendri klausimai

		Visiškai nesutinku	Nesutinku	Nei sutinku, nei nesutinku	Sutinku	Visiškai sutinku
1	Aš žinau, kad nuo onkologinės ligos pasveiksta 80 proc. vaikų.	1	2	3	4	5
2	Aš žinau, kad onkologinės ligos gydymasgali pakenkti mano vaiko vaisingumui – t. y. galimybei susilaukti vaikų baigus gydymą.	1	2	3	4	5
3	Turėjau galimybę aptarti vaisingumo klausimus su medicinos personalu (gydytojais, slaugytojomis, psichologe).	1	2	3	4	5

4	Turėjau pats(-i) paprašyti suteikti informaciją apie vaisingumą.	1	2	3	4	5
5	Informaciją apie vaisingumą gavau ne iš medikų (interneto, bendraujant su kitais tėvais ir pan.).	1	2	3	4	5
6	Aš žinau, kokia nevaisingumo rizika kilo mano vaikui dėl ligos gydymo.	1	2	3	4	5

Klausimai, susiję su pokalbiu su gydytoja onkohematologe

		Visiškai nesutinku	Nesutinku	Nei sutinku, nei nesutinku	Sutinku	Visiškai sutinku
7	Gydytoja onkohematologė minėjo galimą vaisingumo pažeidimą ir jo riziką mano vaikui	1	2	3	4	5
8	Pokalbių metu pateikta informacija apie vaisingumą buvo suprantama ir aiški.	1	2	3	4	5
9	Man buvo pateikta pagalbinė medžiaga apie vaisingumą (lankstinukai, knygos, nuorodos internete ar kt.)	1	2	3	4	5
10	Pateikta pagalbinė medžiaga buvo suprantama ir aiški.	1	2	3	4	5
11	Galėjau įsiterpti ir išsakyti savo nuomonę pokalbiųų dėl vaisingumo metu.	1	2	3	4	5
12	Manau, kad pokalbiai dėl vaisingumo įvyko tinkamu metu.	1	2	3	4	5
13	Manau, kad pokalbiai dėl vaisingumo įvyko tinkamoje aplinkoje.	1	2	3	4	5
14	Manau, kad gydytojai apie vaisingumą kalbėjo tinkamu tonu.	1	2	3	4	5
15	Gydytojai buvo atviri apie tai, ko galima tikėtis iš vaisingumo priežiūros.	1	2	3	4	5
16	Galėjau priimti sprendimą dėl savo vaiko vaisingumo išsaugojimo gydymo.	1	2	3	4	5
17	Man trūko svarbių dalykų per pokalbius dėl vaisingumo.	1	2	3	4	5
18	Aš vis dar turiu klausimų apie vaisingumą.	1	2	3	4	5
19	Jeigu turėsiu klausimų apie vaisingumą ateityje, žinau, kur kreiptis.	1	2	3	4	5

Klausimai po pokalbio apie vaisingumą

		Visiškai nesutinku	Nesutinku	Nei sutinku, nei nesutinku	Sutinku	Visiškai sutinku
20	Aš dabar žinau pakankamai apie vaisingumą.	1	2	3	4	5
21	Aš žinau, kokios yra mano vaiko vaisingumo išsaugojimo galimybės.	1	2	3	4	5
22	Aš žinau apie vaisingumo išsaugojimo gydymo privalumus.	1	2	3	4	5
23	Aš žinau apie vaisingumo išsaugojimo gydymo trūkumus.	1	2	3	4	5
24	Man svarbu, kad mano vaikas ateityje galėtų būti mama/tėčiu.	1	2	3	4	5
25	Aš pykstu, nes sumažėjo mano vaiko galimybės turėti vaikų	1	2	3	4	5
26	Man liūdna, nes sumažėjo mano vaiko galimybės turėti vaikų	1	2	3	4	5
27	Aš galiu atvirai kalbėti apie susirūpinimą dėl savo vaiko vaisingumo.	1	2	3	4	5

Ar yra dalykų, kurių jums trūko per pokalbius ar konsultacijas dėl vaisingumo?

Kaip manote, kada yra tinkamiausias laikas pokalbiui apie vaisingumą?

Kaip manote, kas praėjo gerai per Jūsų vaiko vaisingumo priežiūrą?

Ar turite kitų pastebėjimų ar patarimų dėl vaisingumo priežiūros?

Ačiū už skirtą laiką!

This project has received funding from the European Union's Horizon 2020 – Work Programme on Spreading Excellence and Widening Participation under grant agreement No 952438. "

Table S3: English questionnaire for patients counseled by a fertility specialist.

Twinning in Research and Education to improve survival in Childhood Solid Tumours in Lithuania

TREL

Ver 4.0 2021-06-25

Vilnius University Hospital Santaros Klinikos is participating in Horizon 2020 project „Twinning in Research and Education to improve survival in Childhood Solid Tumours in Lithuania (TREL) Nr.952438".The aim of the project – to improve outcomes and quality of life of children diagnosed with cancer.

After the cancer treatment your child's fertility could be impaired. One of the aims of the project – to evaluate and improve the quality of fertility counseling.We would like to use this questionnaire to find out how you experienced the counseling. I am: (x)

Mother of child ☐ Father of child ☐

I raise:

Girl ☐ Boy ☐

Your child risk for infertility after childhood cancer treatment:

Low ☐ High ☐ I don't know ☐

Statements below are related with fertility counseling. Please indicate to what extent you agree or disagree with these statements by circling one number next to the answer that applies to you (1 – completely disagree, 5 – strongly agree).

General questions:

		Completely disagree	Disagree	Neutral	Agree	Fully agree
1	My doctor had mentioned fertility at the first consult when the diagnosis of my child was discussed.	1	2	3	4	5
2	When I heard the diagnosis and treatment I was worried about my child's fertility.	1	2	3	4	5
3	I had to ask for information about fertility myself.	1	2	3	4	5

Questions about the first conversation regarding fertility with the pediatric oncologist

		Completely disagree	Disagree	Neutral	Agree	Fully agree
4	I felt it was important at that time to receive the information about fertility.	1	2	3	4	5
5	I thought that the moment this was discussed was a good one.	1	2	3	4	5
6	I received supportive material on fertility (leaflets, books, website links etc.)	1	2	3	4	5

This project has received funding from the European Union's Horizon 2020 – Work Programme on Spreading Excellence and Widening Participation under grant agreement No 952438. "

7	I thought the supporting material used during the explanation was clarifying.	1	2	3	4	5
8	The information about infertility was comprehensive and clear.	1	2	3	4	5
9	I missed important things during the conversation.	1	2	3	4	5

Questions regarding the counseling with the gynaecologist/urologist

		Completely disagree	Disagree	Neutral	Agree	Fully agree
10	I think the moment of fertility counseling was a good one.	1	2	3	4	5
11	I received supportive material on fertility (leaflets, books, website links etc.)	1	2	3	4	5
12	I thought the supporting material useddduring the explanation was clarifying.	1	2	3	4	5
13	I know the risk of infertility after treatment.	1	2	3	4	5
14	I was told what treatment options are available to maintain fertility.	1	2	3	4	5
15	The benefits of fertility preservation treatments were discussed.	1	2	3	4	5
16	The disadvantages of fertility preservation treatments were discussed.	1	2	3	4	5
17	I had control over my child's future fertility.	1	2	3	4	5
18	The information about infertility was comprehensive.	1	2	3	4	5
19	The different treatment options were discussed with me.	1	2	3	4	5
20	The information about the treatment optionswas comprehensive.	1	2	3	4	5
21	My caregivers were honest and clear aboutwhat we could expect from the care.	1	2	3	4	5
22	I missed important things during the counseling.	1	2	3	4	5
23	I still have questions about fertility after the counseling.	1	2	3	4	5
24	If I have questions about fertility in the future, I know how to request another counseling.	1	2	3	4	5
25	Decision-making was shared with me, concerning my child treatment.	1	2	3	4	5
26	I am choosing without pressure from others	1	2	3	4	5

Questions after both conversations regarding fertility

		Completely disagree	Disagree	Neutral	Agree	Fully agree
27	I am well informed about fertility.	1	2	3	4	5
28	I now know enough about fertility.	1	2	3	4	5
29	I know the risk of infertility from mychild's treatment.	1	2	3	4	5
30	I know which options are available to maintain my child's fertility.	1	2	3	4	5
31	I know the benefits of fertility preservation treatments.	1	2	3	4	5

This project has received funding from the European Union's Horizon 2020 – Work Programme on Spreading Excellence and Widening Participation under grant agreement No 952438. "

32	I know the disadvantages of fertility preservation treatments.	1	2	3	4	5
33	I am angry because my child's possibilities to have children has been impaired.	1	2	3	4	5
34	I think it's important that my child can be a parent in the future.	1	2	3	4	5
35	I can talk openly about my concerns concerning my child's fertility.	1	2	3	4	5
36	I am sad because my child's possibilitiesto have children has been impaired.	1	2	3	4	5
37	I made the right decision about fertility preservation.	1	2	3	4	5
38	I regret the decision I made.	1	2	3	4	5
39	I would go for a different choice if I had to do it over again.	1	2	3	4	5

Are there things you missed during the first conversation regarding fertility or counseling?

What time do you think is the best time to have a conversation about fertility?

This project has received funding from the European Union's Horizon 2020 – Work Programme on Spreading Excellence and Widening Participation under grant agreement No 952438. "

What do you think went well during fertility care?

Do you have any other additions or tips for fertility care?

Thank you for your time!

This project has received funding from the European Union's Horizon 2020 – Work Programme on Spreading Excellence and Widening Participation under grant agreement No 952438. "

Table S4: English questionnaire for patients informed by a pediatric oncologist only.

Twinning in Research and Education to improve survival in Childhood Solid Tumours in Lithuania

TREL

Ver. 3.0 2021-06-25

Vilnius University Hospital Santaros Klinikos is participating in Horizon 2020 project „Twinning in Research and Education to improve survival in Childhood Solid Tumours in Lithuania (TREL) Nr.952438". The aim of the project – to improve outcomes and quality of life of children diagnosed with cancer.

After the cancer treatment your child's fertility could be impaired. One of the aims of the project – to evaluate and improve the quality of fertility counseling. We would like to use this questionnaire to find out how you experienced the counseling. I am: (x)

Mother of child ☐ Father of child ☐

I raise:

Girl ☐ Boy ☐

Your child risk for infertility after childhood cancer treatment:

Low ☐ Intermediate ☐ High ☐ I don't know ☐

Statements below are related with fertility counseling. Please indicate to what extent you agree or disagree with these statements by circling one number next to the answer that applies to you (1 – completely disagree, 5 – strongly agree).

General questions

		Completely disagree	Disagree	Neutral	Agree	Strongly agree
1	I know that 80 percent of children diagnosed with childhood cancer are curable.	1	2	3	4	5
2	I know that childhood cancer treatment could affect my child's fertility (a possibility to have children after the treatment).	1	2	3	4	5
3	I had a possibility to discuss questions regarding fertility with healthcare personnel (doctors, nurses, psychologist).	1	2	3	4	5
4	I had to ask information about fertility myself.	1	2	3	4	5
5	I got information about fertility not from healthcare personnel but from other sources (Internet, other parents etc.)	1	2	3	4	5
6	I know the risk for my child's infertility after the treatment.	1	2	3	4	5

This project has received funding from the European Union's Horizon 2020 – Work Programme on Spreading Excellence and Widening Participation under grant agreement No 952438. "

Questions regarding conversation with pediatric oncologist:

		Completely disagree	Disagree	Neutral	Agree	Strongly agree
7	Pediatric oncologist mentioned a possibility and risk for fertility impairment for my child.	1	2	3	4	5
8	Information regarding fertility was understandable and clear.	1	2	3	4	5
9	I received supportive material on fertility (leaflets, books, website links etc.)	1	2	3	4	5
10	I thought the supporting material used during the explanation was clarifying.	1	2	3	4	5
11	There was room for me to have a say during a conversation about my child's fertility.		2	3	4	5
12	I thought that the moment this was discussed was a good one.	1	2	3	4	5
13	I think the space was suitable for fertility discussion.	1	2	3	4	5
14	I think practitioners used suitable voice tone during fertility discussion.	1	2	3	4	5
15	My caregivers were honest and clear about what we could expect from the care.	1	2	3	4	5
16	Decision-making was shared with me, concerning my child's treatment	1	2	3	4	5
17	I missed important things during the conversation.	1	2	3	4	5
18	I still have questions about fertility after the conversation.	1	2	3	4	5
19	If I have questions about fertility in the future, I know how to request another conversation.	1	2	3	4	5

This project has received funding from the European Union's Horizon 2020 – Work Programme on Spreading Excellence and Widening Participation under grant agreement No 952438. "

Questions regarding conversation with pediatric oncologist:

		Completely disagree	Disagree	Neutral	Agree	Strongly agree
20	I now know enough about fertility.	1	2	3	4	5
21	I know which options are available to maintain my child's fertility.	1	2	3	4	5
22	I know the benefits of fertility preservation treatment.	1	2	3	4	5
23	I know the disadvantages of fertility preserving treatment.	1	2	3	4	5
24	I think it's important that my child can be a parent in the future.	1	2	3	4	5
25	I am angry because my child's possibilities to have children has been impaired	1	2	3	4	5
26	I am sad because my child's possibilities to have children has been impaired.	1	2	3	4	5
27	I can talk openly about my concerns concerning my child's fertility.	1	2	3	4	5

Are there things you missed during the conversations regarding fertility or counseling?

This project has received funding from the European Union's Horizon 2020 – Work Programme on Spreading Excellence and Widening Participation under grant agreement No 952438. "

What time do you think is the best time to have a conversation about fertility?

What do you think went well during fertility care?

Do you have any other additions or tips on fertility care?

Thank you for your time!

This project has received funding from the European Union's Horizon 2020 – Work Programme on Spreading Excellence and Widening Participation under grant agreement No 952438. "

Table S5: Gonadal damage risk stratification tool (triage)*.

Tumor	Protocol	Treatment arm	CED mg/m^2**	Female infertility risk by CED***	Male infertility risk by CED****
**ematologic malignancies**					
**Acute lymphoblastic leukemia**	Interfant06	Germline LR/rearranged MR HR - SCT	3000	Low	Low
		Rearranged MR HR + SCT	3000 + SCT	High	High
	Interfant21	MR-Low	3000	Low	Low
		MR-High	1000	Low	Low
		HR	SCT	High	High
	EsPhALL	Arm A	9000	High	High
		Arm B	3000	Low	Low
		High risk arm	3976 + SCT	High	High
		High risk arm	5976 + SCT	High	High
	ALLTogether	R1 standard, experimental	0	Low	Low
		R2 standard, Exp arm A, Exp arm B	3000	Low	Low
		R3 standard, Exp InO: IR-high risk	2000	Low	Low
		ABL HR allo-SCT (≥ 1-3 NOPHO blocks)	2000 + SCT	High	High
		ABL IR-High	2000	Low	Low
		HR BCP SCT 3 blocks	4200 + SCT	High	High
		HR BCP chemo 6 blocks	7400	High	High
		HR T-cell without Nelarabine + HR blocks	4200	Low	High
		HR T-cell with Nelarabine single	1000	Low	Low
		HR T-cell with Nelarabine single + HR blocks	3200	Low	Low
		HR T-cell with Nelarabine addition	2000	Low	Low
		HR T-cell with Nelarabine addition + HR blocks	4200	Low	High
		DS-SR	1000	Low	Low
		DS-IR, DS-HR	3000	Low	Low
**LCH (Langerhans Cell Histiocytosis)**	LCH IV	Stratum 1 group 1 (MS-LCH) arm A / B / C / D	0	Low	Low
		Stratum 1 group 2 (SS-LCH)	0	Low	Low
		Stratum 2	0	Low	Low
		Stratum V without clinical neurodegeneration	0	Low	Low
		Stratum V with clinical neurodegeneration	0	Low	Low
**Hodgkin lymphoma**	EuroNet-PHL-C2	TL1	1000	Low	Low
		TL2	2000	Low	Low
		TL2 intensified	2500	Low	Low
		TL3	4000	Low	High
		TL3 intensified	5000	Low	High
**Non-B NHL (Non-Hodgkin Lymphoma)**	Euro LB-02	T-Cell LL stage I-II	2000	Low	Low
		T-Cell LL stage III-IV	3000	Low	Low
		Non-T-Cell LL stage I-II	2000	Low	Low
		Non-T-Cell LL stage III-IV	3000	Low	Low
**LBL (Lymphoblastic lymphoma)**	EICNHL	Standard risk group, I ir II stages pB-LBL	2000	Low	Low
		High risk group, III and IV stages pB-LBL ir T-LBL (all stages)	3000	Low	Low
**B-NHL/B-ALL**	NHL-BFM	R1	1976	Low	Low

**(B-cell Non-Hodgkin Lymphoma/**		R2	4352	Low	High
**acute lymphoblastic leukemia)**		R3-R4-CNS-	4352	Low	High
		R4-CNS+	4352	Low	High
	EICNHL	A	3000	Low	Low
		B I	3300	Low	Low
		B II (High risk)	3300	Low	Low
		C1	5800	Low	High
		C3	5800	Low	High
	Inter-B-NHL ritux	Group B HR	3300	Low	Low
		Group C1	5800	Low	High
		Group C3	5800	Low	High
		PMLBL	4500	Low	High
**Anaplastic large**	EICNHL	Isolated skin ALCL	0	Low	Low
**cell lymphoma (ALCL)**		Systemic ALCL: completely resected	3352	Low	High
		Systemic ALCL: all other stages	6328	High	High
		Patients with CNS+	4352	Low	Low
	ALCL	LR	3352	Low	Low
		SR arm 1 SR arm 3, HR arm 1, HR arm 2, HR arm 3, HR arm 4	6328	High	High
**Acute myelod leukemia**	Nopho DBH AML 2012	SR	0	Low	Low
		HR	0+SCT	High	High
**Acute promyelocytic**	ICC APL 01	SR MRD- / SR MRD+ / HR	0	Low	Low
**leukemia**	ICC APL 02	SR, HR	0	Low	Low
**Solid tumours**					
**Neuroblastoma**	SIOPEN European Low and Intermediate Risk Neuroblastoma (LINES)	LR group 1	0	Low	Low
		LR group 2 2xVP/CARBO	0	Low	Low
		LR group 2 2xVP/CARBO +2x CADO	3000	Low	Low
		LR group 3 4x VP/CARBO	0	Low	Low
		LR group 3 2x VP/CARBO + 2x CADO	3000	Low	Low
		LR group 4	0	Low	Low
		LR group 5 2xVP/CARBO	0	Low	Low
		LR group 5 2xVP/CARBO +2x CADO	3000	Low	Low
		LR group 6 4x VP/CARBO	0	Low	Low
		LR group 6 2x VP/CARBO + 2x CADO	3000	Low	Low
		IR group 7 4x VP/CARBO	0	Low	Low
		IR group 7 2x VP/CARBO + 2x CADO	3000	Low	Low
		IR group 8 2x VP/CARBO + 2x CADO + 1xVP/CARBO + 1xCADO	4500	Low	High
		IR group 8 2x VP/CARBO + 2x CADO + 2xCADO	6000	High	High
		IR group 9 2x VP/CARBO + 2x CADO + 1xVP/CARBO + 1xCADO	4500	Low	High
		IR group 10 4x VP/CARBO	0	Low	Low
		IR group 10 2x VP/CARBO + 2xCADO	3000	Low	Low
		IR group 10 2x VP/CARBO + 4x CADO	6000	High	High
		IR group 10 4x VP/CARBO + 4x CADO	6000	High	High
	HR-NBL-1.8/SIOPEN	BuMel any weight	>12000	High	High
**Ewing**	Ewing 2008	R1 female	25176	High	High
		R3	25176	High	High
		R3 + TreoMel or BuMel	30776	High	High
	Ewing 2012	Arm A R2 VAC	25140	High	High
		BuMel any weight	>23000	High	High
		Arm A R2 VAI	24888	High	High

		Arm B R2 IEVC	23772	High	High
		Arm B R2 BuMel any weight	>23000	High	High
**Osteosarcoma**	EURAMOS 1	MAP	0	Low	Low
		MAPIE	14640	High	High
**Renal tumours**	UMBRELLA 2016/SIOP 2001	AV + AVD, AV + AV1, AV + AV2	0	Low	Low
		AV + HR	8100	High	High
Rhabdoid tumors	EpSSG NRSTS 2005	Cyclophosphamide	17000	High	High
of the kidney	EURHAB <18 mo	3x DOX, 3x ICE, 3xVCA	8892	High	High
(RTK) or of soft	EURHAB <18 mo HD	2x DOX, 2x ICE, 2x VCA + CARBO Thiotepa	50928	High	High
tissue (MRT)	EURHAB >18 mo	3x DOX, 3x ICE, 3xVCA + RT	8892	High	High
	EURHAB >18 mo HD	2x DOX, 2x ICE, 2x VCA + CARBO Thiotepa + RT	50928	High	High
NRSTS*	EpSSG NRSTS 2005	3x ifosfamide	6588	High	High
(Non-Rhabdomyosarcoma Soft		4x ifosfamide	8784	High	High
Tissue Sarcoma)		5x ifosfamide	10980	High	High
		6x ifosfamide	13176	High	High
**Soft tissue sarcomas**	EpSSG RMS 2005	LR subgroup A	0	Low	Low
		SR subgroup B	5800	Low	High
		SR subgroup C (9x Ifosfamide)	13176	High	High
		SR subgroup C (5xIfosfamide)	7320	High	High
		SR subgroup D (9x Ifosfamide)	13176	High	High
		HR ir group A + group C	13176	High	High
		HR ir group A + group D	17376	High	High
		HR ir group B + group C	13176	High	High
		HR ir group B + group D, VHR	17376	High	High
	CWS-guidance-2014	RMS Low risk subgroup A	0	Low	Low
		RMS standard risk subgroup B	5856	Low	High
		RMS standard risk subgroup C without RT	13176	High	High
		RMS standard risk subgroup C with RT	7320	High	High
		RMS standard risk subgroup D	13176	High	High
		RMS High risk subgroups E, F, G	13176	High	High
		RMS very High risk subgroup H	13176	High	High
		"RMS-like" (SySa, STET (EES, pNET), UDS)	13176	High	High
		"Non-RMS-like" (NRTS) Low risk	0	Low	Low
		"Non-RMS-like" (NRTS) Intermediate risk	0	Low	Low
		"Non-RMS-like" (NRTS) High risk	13176	High	High
		Metastatic soft tissue tumours	13176	High	High
		CWS-proposal for angiosarcoma	6000	High	High
**Germ cell tumours**	SIOP CNS GCT II	CCLG	4392	Low	High
		NGGCT	7320	High	High
		2xPEI+2X HD-PEI (High risk non-germinoma)	8540	High	High
	PEB+JEB blocks	Low risk	0	Low	Low
		Intermediate risk	0 (cis/carboplatin)	Low	Low
		High risk	0 (cis/carboplatin)	Low	Low
**Hepatocellular**	PHITT	Group A1 very Low risk HB	0	Low	Low
**carcinoma**		Group A2 very Low risk HB	0 (cisplatin)	Low	Low
		Group B1 Low risk HB / B2	0 (cisplatin)	Low	Low

		Group C Intermediate risk SIOPEL3HR / C5VD/ CDDP-M	0 (cisplatin)	Low	Low
		Group D1 High risk HB SIOPEL4, D2 High risk HB CDCE, CDVI	0 (cis/carboplatin)	Low	Low
		Group E1 resected HCC	0	Low	Low
		Group E2 resected HCC PLADO	0 (cisplatin)	Low	Low
		Group F not resected/metastatic PLADO sorafenib, GEMOX	0 (cisplatin)	Low	Low
**Brain tumours**					
**Optic glioma**	SIOP LGG 2004	Vincristine, carboplatin, etoposide (if allergy: cyclo)	0	Low	Low
**Intradural-extramedulary tumor**	HIT-MED + SCT		49500	High	High
**Medulloblastoma**	PNET 5	MB-SR / MB-WNT-HR(>16years)	17600	High	High
		MB-WNT-HR (<16years)	13200	High	High
		MB-SHH-TP53: no alkylating agents	0	Low	Low
	SR ACNS0331	Cyclophosphamide, lomustine	13200	High	High
	HR ACNS0332	cyclophosphamide	12000	High	High
	HIT-MED Guidance	3x SKK	7200	High	High
		3x SKK+ 2x mSKK	12000	High	High
		3x intensified induction + 1st HDCT + 2nd HDCT + 6x maintenance	4500+intensified induction	High	High
		RT + 8x maintenance	0 (cisplatin)	Low	Low
		2x SKK + RT + 4x maintenance	4800	Low	High
**AT/RT (Atypical teratoid/rhabdoid tumors)**	EURHAB <18 mo	3x DOX, 3x ICE, 3xVCA	8892	High	High
	EURHAB <18 mo HD	2x DOX, 2x ICE, 2x VCA + CARBO Thiotepa	50928	High	High
	EURHAB >18 mo	3x DOX, 3x ICE, 3xVCA + RT	8892	High	High
	EURHAB >18 mo HD	2x DOX, 2x ICE, 2x VCA + CARBO Thiotepa + RT	50928	High	High
**Ependymoma**	HIT-MED Guidance	3x SKK+2x mSKK	12000	High	High
		Local RT	0	Low	Low
		2x mSKK + RT	4800	Low	High
**Pineoblastoma**	HIT-MED Guidance	CARBO/ETO-96h induction + 1st HDCT + 2nd HDCT + 6x maintenance)	4500	Low	High
		RT + 8x maintenance	0 (cisplatin)	Low	Low
		2x SKK + RT + 4x maintenance	4800	Low	High
**High grade glioma, pons glioma**	ACNS0126	Temosolamide	0	Unknown	Unknown
**HGG (High grade glioma)**	Infant HGG 2013/HIT SKK	Elements IIs IIIs/1 IIIs/2 IVs	7200	High	High

The allocated risk is based on the recently published IGHG guidelines [1,2]. The risk allocation of patients with renal tumors is postponed until after surgery, when the definitive treatment including radiotherapy dose is known. For patients with ALL and NHL risk allocation is postponed to the moment of reaching complete remission (CR) or a treatment arm allocation. Some patients with large abdominal tumors at a high risk for infertility will be counseled at a later moment due to the desirability of abdominal surgery with a large abdominal tumor in situ.

* - Amended version of the infertililty risk stratification tool (triage) published by Princess Máxima Center [3].

** - CED (mg/m²) = 1.0 (cumulative cyclophosphamide dose, mg/m²) + 0.244 (cumulative ifosfamide dose, mg/m2) + 0.857 (cumulative procarbazine dose, mg/m²) + 14.286 (cumulative chlorambucil dose, mg/m²) + 15.0 (cumulative BCNU dose, mg/m²) + 16.0 (cumulative CCNU dose, mg/m²) + 40 (cumulative melphalan dose, mg/m²) + 50 (cumulative thiotepa dose, mg/m²) + 100 (cumulative nitrogen mustard dose, mg/m²) + 8.823 (cumulative busulfan dose, mg/m²).

*** - High risk of gonadal damage for females if CED ≥6000 mg/m², ovarian radiotherapy (RT), allo-SCT – oocytes cryopreservation (OC) is strongly recommended, ovarian tissue cryopreservation (OTC) is moderately recommended (2)).

If low-dose alkylating agents (CED <6000 mg/m²), cranial RT, unilateral oophorectomy – OC is moderately recommended [1].

**** - High risk of gonadal damage for males if CED ≥4000 mg/m², testicular RT, allo-SCT – sperm cryopreservation is strongly recommended [2].

If low-dose alkylating agents (CED <4000 mg/m²), cisplatin, orchiectomy, cranial RT – sperm cryopreservation is strongly recommended [2].

References

1. Mulder, R.L.; Font-Gonzalez, A.; Hudson, M.M.; Santen, H.M. van; Loeffen, E.A.H.; Burns, K.C.; Quinn, G.P.; Broeder, E. van D.; Byrne, J.; Haupt, R.; et al. Fertility Preservation for Female Patients with Childhood, Adolescent, and Young Adult Cancer: Recommendations from the PanCareLIFE Consortium and the International Late Effects of Childhood Cancer Guideline Harmonization Group. *The Lancet Oncology* **2021**, *22*, e45–e56, doi:10.1016/S1470-2045(20)30594-5.
2. Mulder, R.L.; Font-Gonzalez, A.; Green, D.M.; Loeffen, E.A.H.; Hudson, M.M.; Loonen, J.; Yu, R.; Ginsberg, J.P.; Mitchell, R.T.; Byrne, J.; et al. Fertility Preservation for Male Patients with Childhood, Adolescent, and Young Adult Cancer: Recommendations from the PanCareLIFE Consortium and the International Late Effects of Childhood Cancer Guideline Harmonization Group. *The Lancet Oncology* **2021**, *22*, e57–e67, doi:10.1016/S1470-2045(20)30582-9.
3. Perk, M.E.M. van der; Kooi, A.-L.L.F. van der; Wetering, M.D. van de; IJgosse, I.M.; Broeder, E. van D.; Broer, S.L.; Klijn, A.J.; Versluys, A.B.; Arends, B.; Ophuis, R.J.A.O.; et al. Oncofertility Care for Newly Diagnosed Girls with Cancer in a National Pediatric Oncology Setting, the First Full Year Experience from the Princess Máxima Center, the PEARL Study. *PLOS ONE* **2021**, *16*, e0246344, doi:10.1371/journal.pone.0246344.

Table S6: Comparison of the original and amended gonadal damage risk stratification tools.

**Original gonadal damage risk stratification tool**					**Amended gonadal damage risk stratification tool**						**Differences from the original tool**
Tumor	Protocol	Treatment arm	CED mg/m^2	Female infertility risk	Tumor	Protocol	Treatment arm	CED mg/m^2	Female infertility risk by CED	Male infertility risk by CED	Males infertility risk by CED was included.
**Hematologic malignancies**					**Hematologic malignancies**						-
Acute Lymphoblastic Leukemia	ALL-11	SR, MR	2000	Low	Acute Lymphoblastic Leukemia	-	-	-	-	-	ALL-11 protocol was removed as it is not used at Vilnius University Hospital Santaros Klinikos (VULSK).
		HR 1-3 +SCT	5600 + SCT	High		-	-	-	-	-	
		HR 1-6 + II	9300	High		-	-	-	-	-	
	Interfant06	Germline LR/rearranged MR HR - SCT	3000	Low		Interfant06	Germline LR/rearranged MR HR - SCT	3000	Low	Low	-
		Rearranged MR HR + SCT	3000+ SCT	High			Rearranged MR HR + SCT	3000 + SCT	High	High	
	-	-	-	-		Interfant21	MR-Low	3000	Low	Low	Interfant 21 protocol was included.
	-	-	-	-			MR-High	1000	Low	Low	
	-	-	-	-			HR	SCT	High	High	
	EsPhALL	Arm A	9000	High		EsPhALL	Arm A	9000	High	High	-
		Arm B	3000	Low			Arm B	3000	Low	Low	
		High risk arm	3976 + SCT	High			High risk arm	3976 + SCT	High	High	
		High risk arm	5976 + SCT	High			High risk arm	5976 + SCT	High	High	
	IntReALL	SR treatment arm A	1976	Low		-	-	-	-	-	InReALL protocol was removed as it is not used at VULSK.
		SR treatment arm A with SCT	1976 + SCT	High		-	-	-	-	-	
		SR treatment arm B	3400	Low		-	-	-	-	-	
		SR treatment arm B with SCT	3400 + SCT	High		-	-	-	-	-	
		HR	1976 + SCT	High			-	-	-	-	
	ALLTogether	R1 standard, experimental	0	Low		ALLTogether	R1 standard, experimental	0	Low	Low	-
		R2 standard, Exp arm A, Exp arm B	3000	Low			R2 standard, Exp arm A, Exp arm B	3000	Low	Low	
		R3 standard, Exp InO: IR-high risk	2000	Low			R3 standard, Exp InO: IR-high risk	2000	Low	Low	
		ABL HR allo-SCT (≥ 1-3 NOPHO blocks)	2000 +SCT	High			ABL HR allo-SCT (≥ 1-3 NOPHO blocks)	2000 + SCT	High	High	
		ABL IR-high	2000	Low			ABL IR-High	2000	Low	Low	
		HR BCP SCT 3 blocks	4200 +SCT	High			HR BCP SCT 3 blocks	4200 + SCT	High	High	
		HR BCP chemo 6 blocks	7400	High			HR BCP chemo 6 blocks	7400	High	High	
		HR T-cell without Nelarabine + HR blocks	4200	Intermediate*			HR T-cell without Nelarabine + HR blocks	4200	Low	High	
		HR T-cell with Nelarabine single	1000	Low			HR T-cell with Nelarabine single	1000	Low	Low	
		HR T-cell with Nelarabine single + HR blocks	3200	Low			HR T-cell with Nelarabine single + HR blocks	3200	Low	Low	
		HR T-cell with Nelarabine addition	2000	Low			HR T-cell with Nelarabine addition	2000	Low	Low	
		HR T-cell with Nelarabine addition + HR blocks	4200	Intermediate*			HR T-cell with Nelarabine addition + HR blocks	4200	Low	High	
		DS-SR	1000	Low			DS-SR	1000	Low	Low	
		DS-IR, DS-HR	3000	Low			DS-IR, DS-HR	3000	Low	Low	
LCH (Langerhans Cell Histiocytosis)	LCH IV	Stratum 1 group 1 (MS-LCH) arm A / B / C / D	0	Low	LCH (Langerhans Cell Histiocytosis)	LCH IV	Stratum 1 group 1 (MS-LCH) arm A / B / C / D	0	Low	Low	-
		Stratum 1 group 2 (SS-LCH)	0	Low			Stratum 1 group 2 (SS-LCH)	0	Low	Low	
		Stratum 2	0	Low			Stratum 2	0	Low	Low	

		Stratum V without clinical neurodegeneration	0	Low			Stratum V without clinical neurodegeneration	0	Low	Low	
		Stratum V with clinical neurodegeneration	0	Low			Stratum V with clinical neurodegeneration	0	Low	Low	
Hodgkin lymphoma	EuroNet-PHL-C2	TL1	1000	Low	Hodgkin lymphoma	EuroNet-PHL-C2	TL1	1000	Low	Low	-
		TL2	2000	Low			TL2	2000	Low	Low	
		TL2 intensified	2500	Low			TL2 intensified	2500	Low	Low	
		TL3	4000	Intermediate*			TL3	4000	Low	High	
		TL3 intensified	5000	Intermediate*			TL3 intensified	5000	Low	High	
Non-B NHL (Non-Hodgkin Lymphoma)	Euro LB-02	T-Cell LL stage I-II	2000	Low	Non-B NHL (Non-Hodgkin Lymphoma)	Euro LB-02	T-Cell LL stage I-II	2000	Low	Low	-
		T-Cell LL stage III-IV	3000	Low			T-Cell LL stage III-IV	3000	Low	Low	
		Non-T-Cell LL stage I-II	2000	Low			Non-T-Cell LL stage I-II	2000	Low	Low	
		Non-T-Cell LL stage III-IV	3000	Low			Non-T-Cell LL stage III-IV	3000	Low	Low	
-	-	-	-	-	LBL (Lymphoblastic lymphoma)	EICNHL	Standard risk group, I ir II stages pB-LBL	2000	Low	Low	EICNHL protocol for LBL (Lymphoblastic lymphoma) was included.
-	-	-	-	-			High risk group, III and IV stages pB-LBL ir T-LBL (all stages)	3000	Low	Low	
B-NHL/B-ALL (B-cell Non-Hodgkin Lymphoma/ acute lymphoblastic leukemia)	SKION B-NHL/B-ALL 2008	Group A	3000	Low	B-NHL/B-ALL (B-cell Non-Hodgkin Lymphoma/ acute lymphoblastic leukemia)	-	-	-	-	-	SKION B-NHL/B-ALL 2008 protocol was removed as it is not used at VULSK.
		Group B	3300	Low		-	-	-	-	-	
		Group C1	6800	High		-	-	-	-	-	
		Group C2	6800	High		-	-	-	-	-	
	-	-	-	-		NHL-BFM	R1	1976	Low	Low	NHL-BFM protocol was included.
	-	-	-	-			R2	4352	Low	High	
	-	-	-	-			R3-R4-CNS-	4352	Low	High	
	-	-	-	-			R4-CNS+	4352	Low	High	
	-	-	-	-		EICNHL	A	3000	Low	Low	EICNHL protocol for B-NHL/B-ALL was included.
	-	-	-	-			B I	3300	Low	Low	
	-	-	-	-			B II (High risk)	3300	Low	Low	
	-	-	-	-			C1	5800	Low	High	
	-	-	-	-			C3	5800	Low	High	
	Inter-B-NHL ritux	Group B HR	3300	Low		Inter-B-NHL ritux	Group B HR	3300	Low	Low	-
		Group C1	5800	Intermediate*			Group C1	5800	Low	High	
		Group C3	5800	Intermediate*			Group C3	5800	Low	High	
		PMLBL	4500	Intermediate*			PMLBL	4500	Low	High	
Anaplastic Large Cell Lymphoma (ALCL)	ALCL	LR	3352	Low	Anaplastic Large Cell Lymphoma (ALCL)	ALCL	LR	3352	Low	Low	-
		SR arm 1 SR arm 3, HR arm 1, HR arm 2, HR arm 3, HR arm 4	6328	High			SR arm 1 SR arm 3, HR arm 1, HR arm 2, HR arm 3, HR arm 4	6328	High	High	
	-	-	-	-		EICNHL	Isolated skin ALCL	0	Low	Low	EICNHL protocol for Anaplastic Large Cell Lymphoma was included.
	-	-	-	-			Systemic ALCL: completely resected	3352	Low	High	
	-	-	-	-			Systemic ALCL: all other stages	6328	High	High	
	-	-	-	-			Patients with CNS+	4352	Low	Low	
Acute Myeloid Leukemia	Nopho DBH AML 2012	SR	0	Low	Acute Myelod Leukemia	Nopho DBH AML 2012	SR	0	Low	Low	-
		HR	0+SCT	High			HR	0+SCT	High	High	
Acute Promyelocytic Leukemia	ICC APL 01	SR MRD- / SR MRD+ / HR	0	Low	Acute Promyelocytic Leukemia	ICC APL 01	SR MRD- / SR MRD+ / HR	0	Low	Low	-
	ICC APL 02	SR, HR	0	Low		ICC APL 02	SR, HR	0	Low	Low	
**Solid tumors**					**Solid tumors**						-
Neuroblastoma	DCOG NBL 2009	OG without N4	0	Low	Neuroblastoma	-	-	-	-	-	DCOG NBL 2009 and DCOG NBL 2009 <1yr protocols were removed as it
		OG with 1x N4	2100	Low		-	-	-	-	-	

		OG with 2x N4	4200	Intermediate*		-	-	-	-	-	is not used at VULSK.
		OG with 3x N4	6300	High		-	-	-	-	-	
		OG with 4x N4	8400	High		-	-	-	-	-	
		MR without N4	10290	High		-	-	-	-	-	
		MR with N4	18690	High		-	-	-	-	-	
		HR without N4	12690	High		-	-	-	-	-	
		HR with N4	21090	High		-	-	-	-	-	
	DCOG NBL 2009 <1yr	OG with 1x N4 <1yr	/kg	Low		-	-	-	-	-	
		OG with 2x N4 <1yr, 3x N4 <1yr, 4x N4 <1yr	/kg	Intermediate*		-	-	-	-	-	
		MR without N4 <1yr, with N4 <1yr	/kg	High		-	-	-	-	-	
		HR without N4 <1yr, with N4 <1yr	/kg	High		-	-	-	-	-	
	-	-	-	-		SIOPEN European Low and Intermediate Risk Neuroblastoma (LINES)	LR group 1	0	Low	Low	SIOPEN European Low and Intermediate Risk Neuroblastoma (LINES) protocol was included.
	-	-	-	-			LR group 2 2xVP/CARBO	0	Low	Low	
	-	-	-	-			LR group 2 2xVP/CARBO +2x CADO	3000	Low	Low	
	-	-	-	-			LR group 3 4x VP/CARBO	0	Low	Low	
	-	-	-	-			LR group 3 2x VP/CARBO + 2x CADO	3000	Low	Low	
	-	-	-	-			LR group 4	0	Low	Low	
	-	-	-	-			LR group 5 2xVP/CARBO	0	Low	Low	
	-	-	-	-			LR group 5 2xVP/CARBO +2x CADO	3000	Low	Low	
	-	-	-	-			LR group 6 4x VP/CARBO	0	Low	Low	
	-	-	-	-			LR group 6 2x VP/CARBO + 2x CADO	3000	Low	Low	
	-	-	-	-			IR group 7 4x VP/CARBO	0	Low	Low	
	-	-	-	-			IR group 7 2x VP/CARBO + 2x CADO	3000	Low	Low	
	-	-	-	-			IR group 8 2x VP/CARBO + 2x CADO + 1xVP/CARBO + 1xCADO	4500	Low	High	
	-	-	-	-			IR group 8 2x VP/CARBO + 2x CADO + 2xCADO	6000	High	High	
	-	-	-	-			IR group 9 2x VP/CARBO + 2x CADO + 1xVP/CARBO + 1xCADO	4500	Low	High	
	-	-	-	-			IR group 10 4x VP/CARBO	0	Low	Low	
	-	-	-	-			IR group 10 2x VP/CARBO + 2xCADO	3000	Low	Low	
	-	-	-	-			IR group 10 2x VP/CARBO + 4x CADO	6000	High	High	
	-	-	-	-			IR group 10 4x VP/CARBO + 4x CADO	6000	High	High	
	-	-	-	-		HR-NBL-1.8/SIOPEN	BuMel any weight	>12000	High	High	HR-NBL-1.8/SIOPEN protocol was included.
Ewing	Ewing 2008	R1 female	25176	High	Ewing	Ewing 2008	R1 female	25176	High	High	-
		R3	25176	High			R3	25176	High	High	
		R3 + TreoMel	30776	High			R3 + TreoMel or BuMel	30776	High	High	
	-	-	-	-		Ewing 2012	Arm A R2 VAC	25140	High	High	Ewing 2012 protocol was included.
	-	-	-	-			BuMel any weight	>23000	High	High	
	-	-	-	-			Arm A R2 VAI	24888	High	High	
	-	-	-	-			Arm B R2 IEVC	23772	High	High	
	-	-	-	-			Arm B R2 BuMel any weight	>23000	High	High	
Osteosarcoma	EURAMOS 1	MAP	0	Low	Osteosarcoma	EURAMOS 1	MAP	0	Low	Low	-
		MAPIE	14640	High			MAPIE	14640	High	High	
Renal tumors	UMBRELLA 2016/SIOP 2001**	AV + AVD, AV + AV1, AV + AV2	0	Low	Renal tumours	UMBRELLA 2016/SIOP 2001	AV + AVD, AV + AV1, AV + AV2	0	Low	Low	-

		AV + HR	8100	High
Rhabdoid tumors of the kidney (RTK) or of soft tissue (MRT)	EpSSG NRSTS 2005	Cyclophosphamide	17000	High
	EURHAB <18 mo	3x DOX, 3x ICE, 3xVCA	8892	High
	EURHAB <18 mo HD	2x DOX, 2x ICE, 2x VCA + CARBO Thiotepa	50928	High
	EURHAB >18 mo	3x DOX, 3x ICE, 3xVCA + RT	8892	High
	EURHAB >18 mo HD	2x DOX, 2x ICE, 2x VCA + CARBO Thiotepa + RT	50928	High
NRSTS (Non-Rhabdomyosarcoma Soft Tissue Sarcoma)	EpSSG NRSTS 2005	3x ifosfamide	6588	High
		4x ifosfamide	8784	High
		5x ifosfamide	10980	High
		6x ifosfamide	13176	High
Soft tissue sarcomas	EpSSG RMS2005	LR subgroup A	0	Low
		SR subgroup B	5800	Intermediate*
		SR subgroup C (9x Ifosfamide)	13176	High
		SR subgroup C (5xIfosfamide)	7320	High
		SR subgroup D (9x Ifosfamide)	13176	high
		HR and group A + group C	13176	high
		HR and group A + group D	17376	High
		HR and group B + group C	13176	High
		HR and group B + group D, VHR	17376	High
	-	-	-	-
	-	-	-	-
	-	-	-	-
	-	-	-	-
	-	-	-	-
	-	-	-	-
	-	-	-	-
	-	-	-	-
	-	-	-	-
	-	-	-	-
	-	-	-	-
	-	-	-	-
	-	-	-	-
Germ cell tumors	SIOP CNS GCT II	-	-	-
		NGGCT	7320	High
		-	-	-
	-	-	-	-
	-	-	-	-

		AV + HR	8100	High	High	
Rhabdoid tumors of the kidney (RTK) or of soft tissue (MRT)	EpSSG NRSTS 2005	Cyclophosphamide	17000	High	High	-
	EURHAB <18 mo	3x DOX, 3x ICE, 3xVCA	8892	High	High	
	EURHAB <18 mo HD	2x DOX, 2x ICE, 2x VCA + CARBO Thiotepa	50928	High	High	
	EURHAB >18 mo	3x DOX, 3x ICE, 3xVCA + RT	8892	High	High	
	EURHAB >18 mo HD	2x DOX, 2x ICE, 2x VCA + CARBO Thiotepa + RT	50928	High	High	
NRSTS* (Non-Rhabdomyosarcoma Soft Tissue Sarcoma)	EpSSG NRSTS 2005	3x ifosfamide	6588	High	High	-
		4x ifosfamide	8784	High	High	
		5x ifosfamide	10980	High	High	
		6x ifosfamide	13176	High	High	
Soft tissue sarcomas	EpSSG RMS 2005	LR subgroup A	0	Low	Low	-
		SR subgroup B	5800	Low	High	
		SR subgroup C (9x Ifosfamide)	13176	High	High	
		SR subgroup C (5xIfosfamide)	7320	High	High	
		SR subgroup D (9x Ifosfamide)	13176	High	High	
		HR ir group A + group C	13176	High	High	
		HR ir group A + group D	17376	High	High	
		HR ir group B + group C	13176	High	High	
		HR ir group B + group D, VHR	17376	High	High	
	CWS-guidance-2014	RMS Low risk subgroup A	0	Low	Low	CWS-guidance-2014 protocol was included.
		RMS standard risk subgroup B	5856	Low	High	
		RMS standard risk subgroup C without RT	13176	High	High	
		RMS standard risk subgroup C with RT	7320	High	High	
		RMS standard risk subgroup D	13176	High	High	
		RMS High risk subgroups E, F, G	13176	High	High	
		RMS very High risk subgroup H	13176	High	High	
		“RMS-like” (SySa, STET (EES, pNET), UDS)	13176	High	High	
		“Non-RMS-like” (NRTS) Low risk	0	Low	Low	
		“Non-RMS-like” (NRTS) Intermediate risk	0	Low	Low	
		“Non-RMS-like” (NRTS) High risk	13176	High	High	
		Metastatic soft tissue tumours	13176	High	High	
		CWS-proposal for angiosarcoma	6000	High	High	
Germ cell tumors	SIOP CNS GCT II	CCLG	4392	Low	High	CCLG and 2xPEI+2xHD-PEI (High risk non-germinoma) treatment arms of SIOP CNS GCT II protocol was included.
		NGGCT	7320	High	High	
		2xPEI+2x HD-PEI (High risk non-germinoma)	8540	High	High	
	PEB+JEB blocks	Low risk	0	Low	Low	PEB + JEB blocks for germ cells tumors were included.
		Intermediate risk	0 (cis/carboplatin)	Low	Low	

	-	-	-	-			High risk	0 (cis/carboplatin)	Low	Low	
Liver tumors Hepatocellular carcinoma	PHITT	Group A1 very low risk HB	0	Low	Liver tumors Hepatocellular carcinoma	PHITT	Group A1 very Low risk HB	0	Low	Low	-
		Group A2 very low risk HB	0 (cisplatin)	Low			Group A2 very Low risk HB	0 (cisplatin)	Low	Low	
		Group B1 Low risk HB / B2	0 (cisplatin)	Low			Group B1 Low risk HB / B2	0 (cisplatin)	Low	Low	
		Group C intermediate risk SIOPEL3HR / C5VD/ CDDP-M	0 (cisplatin)	Low			Group C Intermediate risk SIOPEL3HR / C5VD/ CDDP-M	0 (cisplatin)	Low	Low	
		Group D1 high risk HB SIOPEL4, D2 high risk HB CDCE, CDVI	0 (cis/carboplatin)	Low			Group D1 High risk HB SIOPEL4, D2 High risk HB CDCE, CDVI	0 (cis/carboplatin)	Low	Low	
		Group E1 resected HCC	0	Low			Group E1 resected HCC	0	Low	Low	
		Group E2 resected HCC PLADO	0 (cisplatin)	Low			Group E2 resected HCC PLADO	0 (cisplatin)	Low	Low	
		Group F unresected/metastatic PLADO sorafenib, GEMOX	0 (cisplatin)	Low			Group F not resected/metastatic PLADO sorafenib, GEMOX	0 (cisplatin)	Low	Low	
**Brain tumors**					**Brain tumours**						-
Opticus glioma	SIOP LGG 2004	Vincristine, carboplatin, etoposide. (In case of allergy: cyclo)	0	Low	Opticus glioma	SIOP LGG 2004	Vincristine, carboplatin, etoposide (if allergy: cyclo)	0	Low	Low	-
Intradural-extramedulary tumor	HIT-MED + SCT		49500	High	Intradural-extramedulary tumor	HIT-MED + SCT		49500	High	High	-
Medulloblastoma	SR ACNS0331	Cyclophosphamide, lomustine	13200	High	Medulloblastoma	SR ACNS0331	Cyclophosphamide, lomustine	13200	High	High	-
	HR ACNS0332	cyclophosphamide	12000	High		HR ACNS0332	cyclophosphamide	12000	High	High	-
	PNET 5	MB-SR / MB-WNT-HR(>16years)	17600	High		PNET 5	MB-SR / MB-WNT-HR(>16years)	17600	High	High	-
		MB-WNT-HR (<16years)	13200	High			MB-WNT-HR (<16years)	13200	High	High	
		MB-SHH-TP53: No alkylating agents	0	Low			MB-SHH-TP53: no alkylating agents	0	Low	Low	
	-	-	-	-		HIT-MED Guidance	3x SKK	7200	High	High	HIT-MED Guidance protocol was included.
	-	-	-	-			3x SKK+ 2x mSKK	12000	High	High	
	-	-	-	-			3x intensified induction + 1st HDCT + 2nd HDCT + 6x maintenance	4500+intensified induction	High	High	
	-	-	-	-			RT + 8x maintenance	0 (cisplatin)	Low	Low	
	-	-	-	-			2x SKK + RT + 4x maintenance	4800	Low	High	
AT/RT (Atypical teratoid/rhabdoid tumors)	EURHAB <18 mo	3x DOX, 3x ICE, 3xVCA	8892	High	AT/RT (Atypical teratoid/rhabdoid tumors)	EURHAB <18 mo	3x DOX, 3x ICE, 3xVCA	8892	High	High	-
	EURHAB <18 mo HD	2x DOX, 2x ICE, 2x VCA + CARBO Thiotepa	50928	High		EURHAB <18 mo HD	2x DOX, 2x ICE, 2x VCA + CARBO Thiotepa	50928	High	High	
	EURHAB >18 mo	3x DOX, 3x ICE, 3xVCA + RT	8892	High		EURHAB >18 mo	3x DOX, 3x ICE, 3xVCA + RT	8892	High	High	
	EURHAB >18 mo HD	2x DOX, 2x ICE, 2x VCA + CARBO Thiotepa + RT	50928	High		EURHAB >18 mo HD	2x DOX, 2x ICE, 2x VCA + CARBO Thiotepa + RT	50928	High	High	
Dysgerminoma	WHO IV SIOP CNS GCT II HR-non-germinoma	PEI	8540	High	-	-	-	-	-	-	WHO IV SIOP CNS GCT II HR-non-germinoma protocol was removed as it is not used at VULSK.
-	-	-	-	-	Ependymoma	HIT-MED Guidance	3x SKK+2x mSKK	12000	High	High	HIT-MED Guidance protocol was ependymoma was included.
-	-	-	-	-			Local RT	0	Low	Low	
-	-	-	-	-			2x mSKK + RT	4800	Low	High	
-	-	-	-	-	Pineoblastoma	HIT-MED Guidance	CARBO/ETO-96h induction + 1st HDCT + 2nd HDCT + 6x maintenance)	4500	Low	High	HIT-MED Guidance protocol for pineoblastoma was included.
-	-	-	-	-			RT + 8x maintenance	0 (cisplatin)	Low	Low	
-	-	-	-	-			2x SKK + RT + 4x maintenance	4800	Low	High	
High grade glioma, Pons	ACNS0126	Temozolamide	0	Unknown	High grade glioma, pons	ACNS0126	Temosolamide	0	Unknown	Unknown	-

glioma					glioma							
HGG (High grade glioma)	Infant HGG 2013/HIT SKK	Elements IIs IIIs/1 IIIs/2 IVs	7200	High	HGG (High grade glioma)	Infant HGG 2013/HIT SKK	Elements IIs IIIs/1 IIIs/2 IVs	7200	High	High	-

* - Intermediate risk for girls was removed – according to the newest recommendations the risk for girls is defined as low or high [1].

1. Mulder, R.L.; Font-Gonzalez, A.; Hudson, M.M.; Santen, H.M. van; Loeffen, E.A.H.; Burns, K.C.; Quinn, G.P.; Broeder, E. van D.; Byrne, J.; Haupt, R.; et al. Fertility Preservation for Female Patients with Childhood, Adolescent, and Young Adult Cancer: Recommendations from the PanCareLIFE Consortium and the International Late Effects of Childhood Cancer Guideline Harmonization Group. The Lancet Oncology **2021**, 22, e45–e56, doi:10.1016/S1470-2045(20)30594-5.

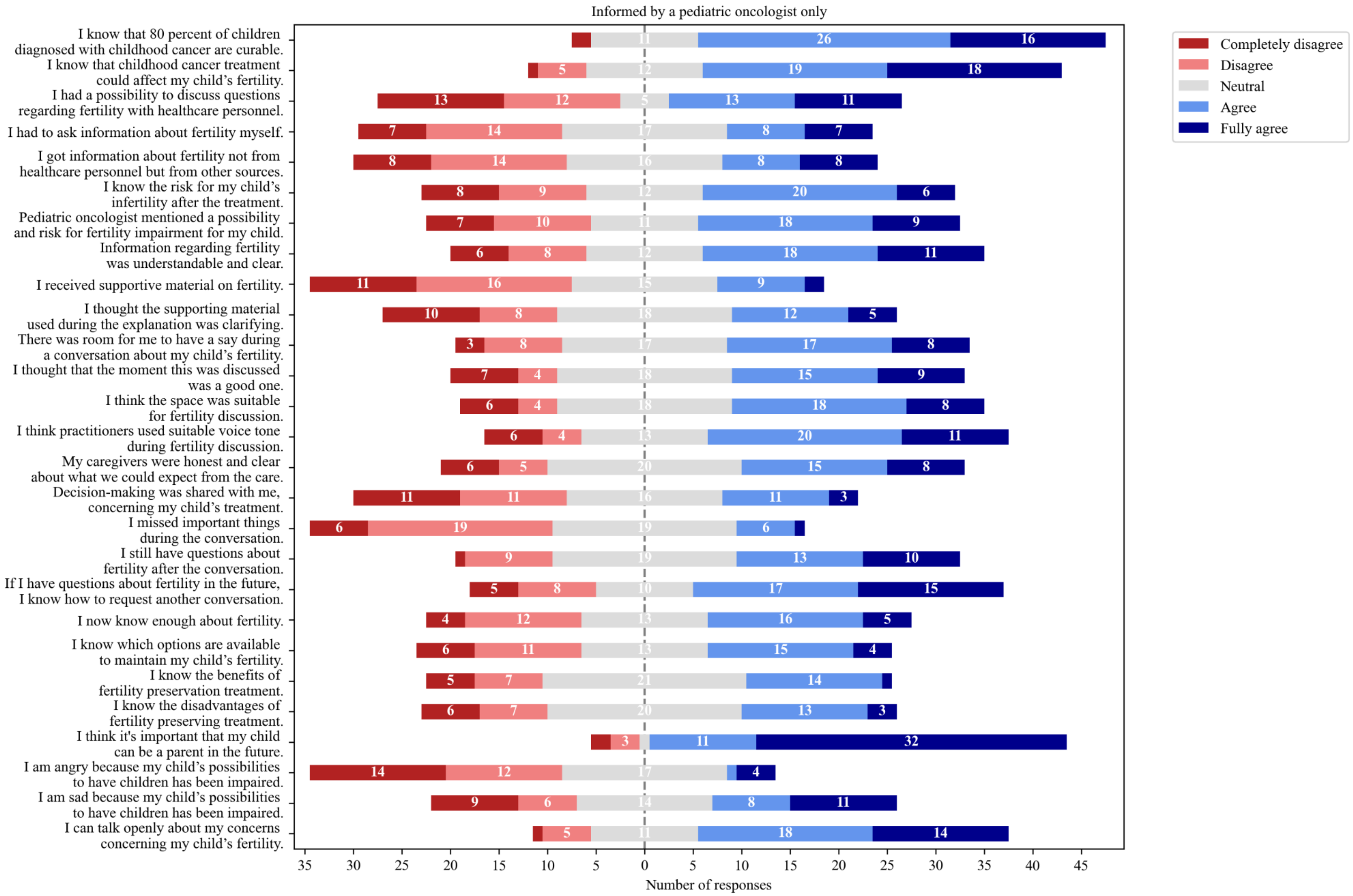

Figure S1: All answers of respondents informed by a pediatric oncologist only (n=55).

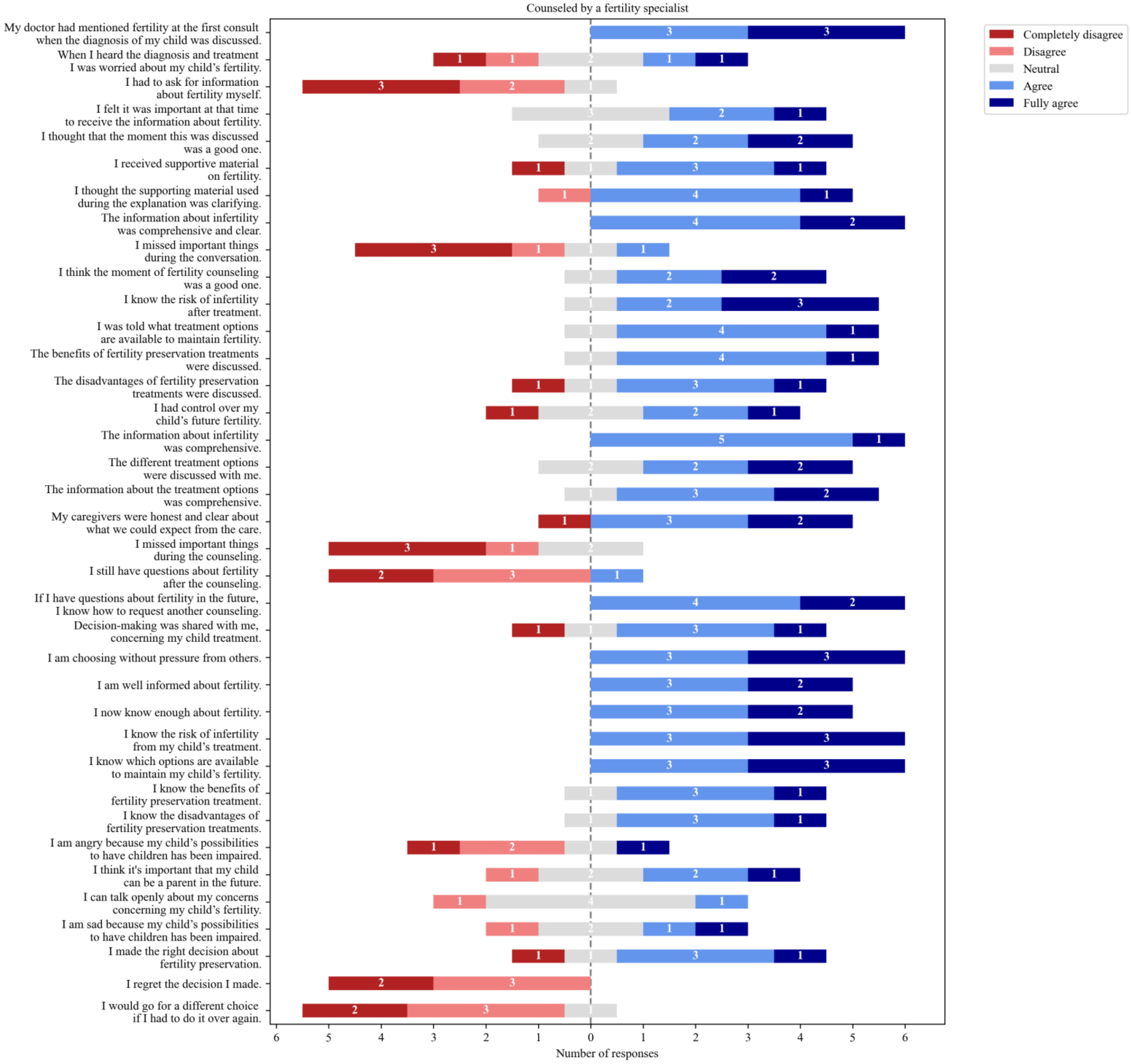

Figure S2: All answers of respondents counseled by a fertility specialist (n=6).
